# A ‘Tangled Web’ in the CNS: unraveling neutrophil extracellular traps in neurological disorders

**DOI:** 10.1186/s13024-026-00967-9

**Published:** 2026-06-24

**Authors:** Haixia Wang, Ruiming Wen, Emily Parker, Luodan Yang

**Affiliations:** 1 Laboratory of Exercise and Neurobiology, School of Physical Education and Sports Science, South China Normal University, Guangzhou, 510006 China; 2 Medical College of Georgia, Augusta University, 1120 15th Street, Augusta, GA 30912 USA

**Keywords:** Neutrophil extracellular traps, Neuroinflammation, Brain disorders, Blood-brain barrier, NETosis, Neuroprotection

## Abstract

Neutrophil Extracellular Traps (NETs) are web-like structures composed of DNA, histones, and antimicrobial proteins released by activated neutrophils, which play a critical role in modulating neutrophil-mediated immune responses. Initially recognized for their role in host defense, NETs function as “molecular traps” that rapidly ensnare pathogens. They then achieve efficient clearance by directly degrading virulence factors through the action of associated antimicrobial proteins. However, the functions of NETs extend beyond immune defense. Dysregulation of NETs in pathological conditions can lead to detrimental effects. Recent studies have highlighted the importance of aberrant NET formation and impaired clearance in the pathogenesis of central nervous system (CNS) diseases, making them a field hotspot. In view of this, we discuss NETs, the structural characteristics and diverse generation patterns of NETs, and clarify how neutrophils cross the blood-brain barrier (BBB) to enter the CNS. We also explore triggers of NETosis within the brain, such as oxidative stress and inflammatory mediators. Furthermore, we analyze the pathological contributions of NETs to a range of CNS disorders, including stroke, traumatic brain injury (TBI), subarachnoid hemorrhage (SAH), Alzheimer’s disease (AD), multiple sclerosis (MS), etc. Finally, we summarize emerging neuroprotective strategies that target NETs, highlighting advances in interventions designed to inhibit NET formation or promote their degradation. By synthesizing current evidence, this review aims to uncover the mysterious veil of NETs in neurological diseases and to offer new insights for understanding disease mechanisms and developing targeted therapeutic approaches.

## Background

Neutrophils, the most prevalent type of white blood cells in human peripheral blood, serve as the initial line of defense in innate immunity. Traditionally, they are recognized for combating infections primarily through phagocytosis, degranulation, and respiratory burst [1, 2]. In 2004, however, Brinkmann and colleagues made a pivotal discovery by identifying a distinct cellular defense mechanism where activated neutrophils released reticular fibrous structures that efficiently trap and kill bacteria. This structure was termed neutrophil extracellular traps (NETs), and the process through which neutrophils release NETs is referred to as NETosis [3].

The discovery of NETs has fundamentally altered the traditional understanding of neutrophil functions. Initially, NETs were characterized as an effective antibacterial defense mechanism capable of physically capturing and efficiently eliminating a wide range of pathogens, including bacteria, fungi, and viruses [3–5]. However, ongoing research has revealed that NETs serve purposes beyond mere defense. Under pathological conditions such as infections, trauma, and autoimmune diseases, abnormal generation or clearance of NETs can result in their excessive accumulation within tissues. This accumulation may subsequently inflict damage on host tissues through their cytotoxic components or by triggering inflammatory responses [6, 7]. For example, NETs can activate synovial cells to secrete the inflammatory factor IL-33, thereby exacerbating joint damage [8]. In the context of cardiovascular diseases, NETs have been shown to promote thrombosis and contribute to the progression of atherosclerosis [9]. These findings indicate that NETs act as a genuine “double-edged sword”, and their functional imbalance is closely associated with the pathological progression of numerous diseases.

Early research on NETs primarily focused on infectious diseases and peripheral inflammatory conditions. However, subsequent studies have demonstrated that this defensive “net” also plays a detrimental role in neurological disorders [10, 11]. Under physiological conditions, neutrophils and NETs are scarcely present in the brain. However, following brain injury, inflammatory factors and chemokines are upregulated, and the blood-brain barrier (BBB) becomes compromised [12]. These changes promote the recruitment of neutrophils from the periphery to the lesion site, where they infiltrate the brain via the damaged BBB or adhere to endothelial cells and migrate into the parenchyma [13]. There, they release NETs, which exacerbate immune inflammation and tissue damage. Thus, NETs transition from protective guards to harmful accomplices. Rapid neutrophil recruitment and substantial NET formation have been observed in nearly all central nervous system (CNS) injury and disease models, including acute conditions such as stroke and TBI, as well as chronic neurodegenerative diseases like AD and Parkinson’s disease [14–17]. These NETs are located in the brain parenchyma, inside blood vessels, or around vascular structures, where they engage in complex interactions with the neurovascular unit. Studies indicate that NETs contribute to CNS injury through multiple mechanisms. The histones and proteases they carry can directly disrupt BBB integrity and increase its permeability [18]. Neurons and glial cells can detect NETs, triggering robust neuroinflammation, endoplasmic reticulum (ER) stress, and neuronal apoptosis [19–21]. Notably, the procoagulant properties of NETs can promote microvascular thrombosis, exacerbating cerebral ischemia [22, 23]. Moreover, NETs have also been implicated in glioma progression through modulation of the immune microenvironment [24, 25]. Collectively, these findings underscore a strong and expanding link between NETs and neurological diseases. A deeper understanding of the specific mechanisms by which NETs operate in these contexts will be crucial for developing novel neuroimmune-targeted strategies.

## Main text

### The structure and composition of NETs

**Fig. 1:**
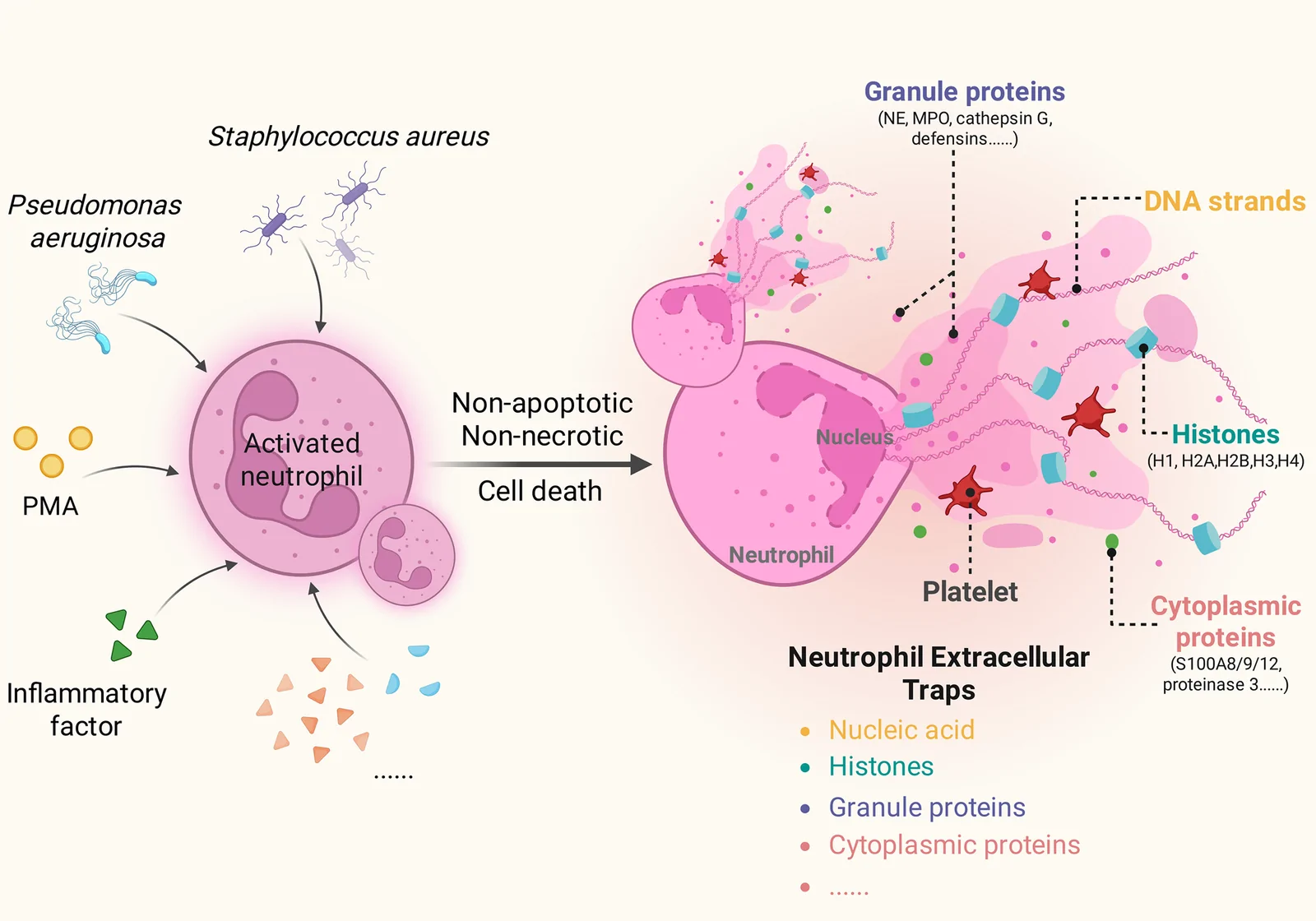
The structure and composition of NETs. Under stimulation by *Staphylococcus aureus, Pseudomonas aeruginosa*, PMA, or inflammatory factors, activated neutrophils undergo non-apoptotic, non-necrotic cell death. Subsequently, they release NETs, which consist of DNA strands, histones (H1, H2A, H2B, H3, H4), granule proteins (NE, MPO, cathepsin G), and cytoplasmic proteins. Notably, NETs also exhibit prothrombotic properties, contributing to thrombus formation

Upon stimulation by pathogens such as *Staphylococcus aureus* or *Pseudomonas aeruginosa*, inflammatory mediators, or experimental NETosis inducers such as phorbol 12-myristate 13-acetate (PMA), neutrophils can initiate the NETosis program. This process culminates in the extracellular release of reticular structures composed of DNA, histones, and antimicrobial proteins, collectively termed NETs [3, 26] (Fig. 1). Fundamentally, NETs are functional complexes formed when neutrophils reorganize and extrude critical intracellular molecules in response to specific stimuli [27]. Their primary constituents include nucleic acids, histones, granular proteins, and minor amounts of cytoplasmic proteins. Together, these components dictate the structural stability of NETs, their capacity to capture pathogens, and their role in modulating inflammation [28].

Chromatin DNA serves as the core backbone of NETs. During the formation of NETs, nucleosome structures depolymerize, and histones, following biochemical modification, dissociate from the DNA. This cascade allows highly compacted chromatin to unfurl into linear DNA strands, which subsequently intertwine to form a reticular network [29]. The integrity of the DNA backbone is paramount; degradation by deoxyribonuclease I (DNase I) destroys the structural framework of NETs, thereby impairing their ability to entrap pathogens [30, 31]. Histones represent a crucial protein constituent, with H1, H2A, H2B, H3, and H4 acting as the primary core elements [32]. During NETosis, histones undergo modifications such as citrullination and acetylation. These alterations reduce their binding affinity for DNA, facilitating chromatin decondensation and subsequent extracellular release [33, 34]. Notably, protein arginine deiminase 4 catalyzes the conversion of histone arginine residues to citrulline, acting as a pivotal regulatory molecule in the formation of NETs [35].

Granular proteins endow NETs with vital antimicrobial capabilities and the capacity to regulate inflammation. These primarily include neutrophil elastase (NE), myeloperoxidase (MPO), cathepsin G, defensins, lactoferrin, lysozyme C, gelatinase, proteinase-3, and calprotectin [36–38]. Upon activation by microbial infections or inflammatory stimuli, neutrophils undergo chromatin decondensation and nuclear envelope disintegration. The subsequent mixing of granular proteins with chromatin culminates in the extracellular extrusion of complexes containing DNA, histones, and granular proteins to assemble NETs [39, 40]. While these structures effectively capture and confine pathogens, under pathological conditions, components such as histones, NE, and MPO can critically amplify inflammation, induce endothelial injury, and promote coagulation cascades.

### The multivariate generation model of NETs

**Fig. 2:**
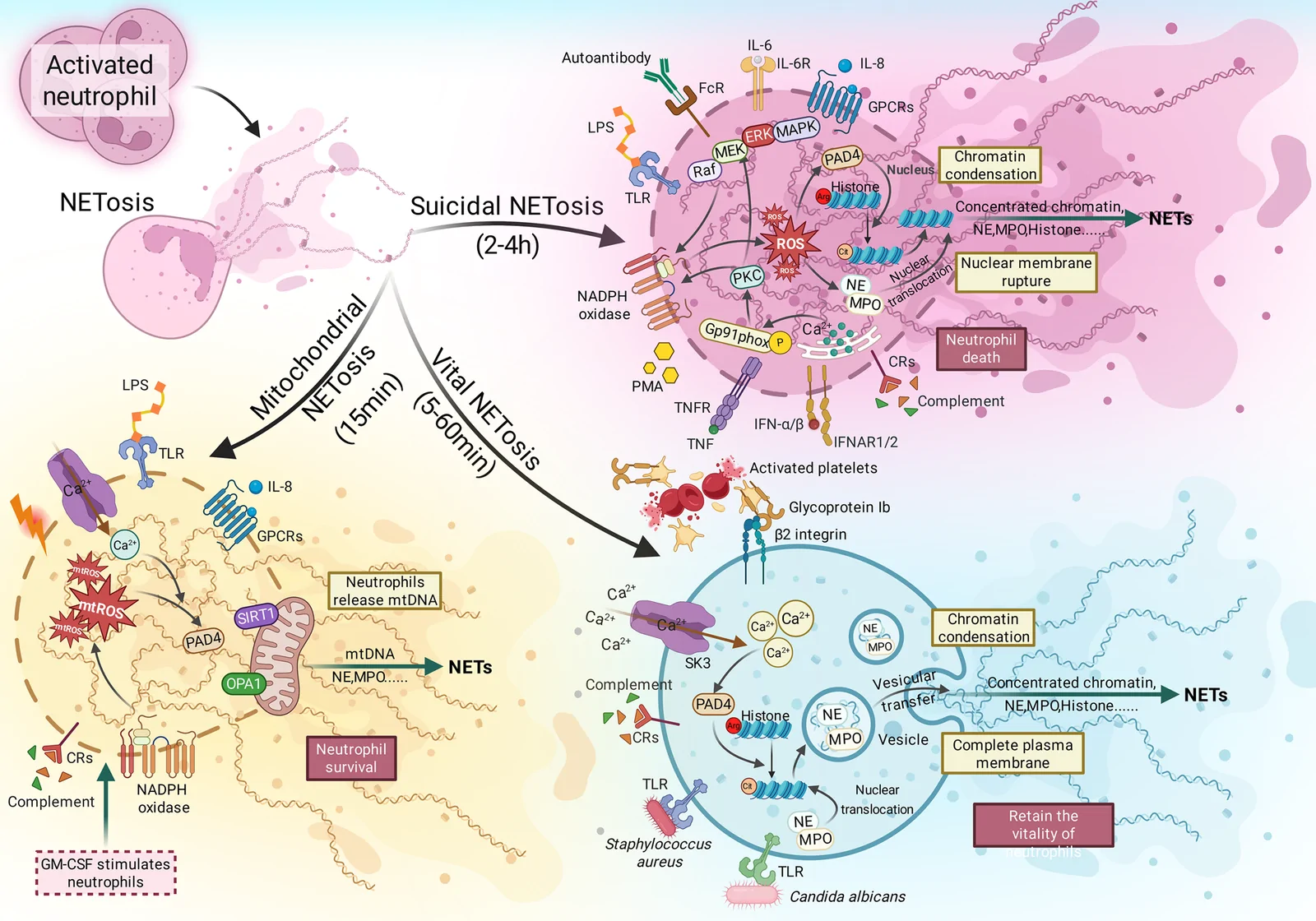
Three pathways for NET formation. In suicidal NETosis, activated neutrophils undergo nuclear membrane rupture upon stimulation, followed by chromatin condensation and NET release, and ultimately cell death; this process takes 2–4 hours. In vital NETosis, neutrophils interact with platelets and pathogens, releasing chromatin via vesicles to form NETs (without ROS involvement), while the cells retain viability; this process lasts 5–60 minutes. In mitochondrial NETosis, stimulated neutrophils release mtDNA and other components from mitochondria to assemble into NETs (resembling suicidal NETosis in mechanism), and the cells remain viable; this process is completed in just 15 minutes. All three pathways are regulated by associated signaling pathways

The formation of NETs does not proceed via a singular mechanism. Rather, this process is predominantly driven by suicidal NETosis, vital NETosis, and mitochondrial DNA (mtDNA)-driven NETosis [28] (Fig. 2). Additionally, recent investigations have identified several novel paradigms, including pyroptosis-associated NETosis [41], autophagy-associated NETosis [42], NETosis induced by endogenous stimuli [43], and transcellular NETosis triggered by interactions between neutrophils and other cells [44]. Nevertheless, these more recently discovered subtypes currently remain in the exploratory stages of research.

Different modes of NETosis likely reflect context-specific neutrophil adaptation rather than redundant pathways. In physiological host defense, vital or mitochondrial NETosis enables rapid extracellular DNA release while partially preserving neutrophil antimicrobial functions, which may support early pathogen containment at mucosal or infectious sites [45]. In contrast, suicidal NETosis is usually induced by stronger or persistent stimuli and produces large chromatin-protein scaffolds at the cost of neutrophil viability [40, 46]. Outside neurological disorders, these programs can become maladaptive in autoimmune disease, thrombosis, sepsis, and cancer by exposing intracellular autoantigens, supporting thrombus formation, amplifying systemic inflammation, or promoting tumor cell dissemination [47–50]. In CNS diseases, platelet-derived HMGB1, complement C5a, and ischemia-associated neutrophil metabolic reprogramming may redirect NETosis toward BBB disruption, endothelial injury, microvascular occlusion, and sustained neuroinflammation [51–54]. Thus, the mode of NETosis is shaped by stimulus intensity, receptor engagement, redox state, calcium signaling, PAD activity, and local NET clearance capacity, which helps explain why NETs can be protective in infection but pathogenic in autoimmune, thrombotic, and neuroinflammatory disorders.

#### Suicidal NETosis

Suicidal NETosis is the best-characterized mode of NET formation and is defined by terminal neutrophil death [55]. It is typically induced by strong stimuli, including bacterial lipopolysaccharide (LPS), PMA, interleukin (IL)-8/6, autoantibodies, tumor necrosis factor (TNF), and interferon (IFN), and usually requires approximately 2–4 hours to complete [56, 57]. Morphologically, activated neutrophils undergo chromatin decondensation, nuclear envelope rupture, mixing of decondensed chromatin with granular proteins, and eventual plasma membrane rupture, leading to the extracellular release of DNA-protein networks that assemble into NETs [40, 45, 46].

Mechanistically, these stimuli activate neutrophils through surface receptors such as Toll-like receptors (TLRs), Fc receptors, G protein-coupled receptors, complement receptors, and cytokine receptors [58–60]. These upstream signals increase cytosolic Ca^2+^ and activate protein kinase C (PKC), Raf-MEK-ERK, p38/MAPK, Rac, and the NADPH oxidase (NOX) complex, resulting in reactive oxygen species (ROS) production [61–66]. ROS act as central mediators by promoting granular protein mobilization and facilitating the translocation of MPO and NE into the nucleus, while also cooperating with Ca^2+^ signaling to activate PAD4 [7]. Within the nucleus, NE cleaves histones and disrupts chromatin packaging, MPO further promotes chromatin decondensation, and PAD4 catalyzes histone citrullination, reducing histone-DNA affinity and accelerating chromatin relaxation [40, 46, 67, 68].

Additional mechanisms contribute to membrane rupture and NET release. Cyclin-dependent kinase 4/6 (CDK4/6) activation may participate in nuclear envelope disruption, whereas caspase-4/5/11 and NE promote Gasdermin D (GSDMD) activation and pore formation in granular and plasma membranes [41, 69–73]. These pores facilitate granular protein release and chromatin extrusion. Ultimately, decondensed chromatin combines with cytoplasmic and granular proteins and is expelled through membrane permeabilization and cell lysis, producing NETs at the cost of neutrophil viability.

#### Vital NETosis

Unlike suicidal NETosis, vital NETosis preserves neutrophil phagocytosis and chemotaxis [74]. This process completes within 5 to 60 minutes and operates independently of ROS and NOX [75]. It initiates when neutrophils detect specific stimuli. Signals like platelets, complement C3, Staphylococcus aureus, or Candida albicans activate surface TLR2/4 [57, 76–78], complement receptors, or platelet glycoprotein Ib-neutrophil β2 integrin complexes [79, 80]. This activation opens SK3 potassium channels, promoting Ca^2+^ influx [81]. Elevated Ca^2+^ activates PAD4 to citrullinated histone H3 (H3cit). This modification weakens electrostatic bonds between histones and DNA, triggering chromatin decondensation [82, 83]. The nucleus then undergoes morphological expansion and blebbing. It forms vesicles that encapsulate the chromatin, which subsequently merge with cytoplasmic granules [46]. Finally, these vesicles fuse with the plasma membrane, releasing chromatin and granular proteins extracellularly to assemble NETs. Throughout this cascade, the nuclear and plasma membranes remain structurally intact [28]. Nevertheless, its precise molecular mechanisms require further elucidation.

#### Mitochondrial NETosis

Mitochondrial DNA (mtDNA)-driven NETosis incorporates mitochondrial instead of nuclear DNA into the NET backbone. This rapid process concludes within 15 minutes [84]. Priming neutrophils with granulocyte/macrophage colony-stimulating factor (GM-CSF) allows Toll-like receptor 4 (TLR4) to detect LPS. Alternatively, complement component 5a (C5a) receptors bind their specific ligands [84]. These signals drive eighty percent of neutrophils to form NETs containing exclusively mtDNA [84]. Upon activation, the NOX complex generates primary ROS. Concurrently, mitochondrial complexes I and III leak electrons to produce mitochondrial ROS. This creates a synergistic oxidative environment [85, 86]. Simultaneously, calcium-activated small conductance potassium channels modulate intracellular calcium to facilitate subsequent steps [87]. Conversely, NOX inhibitors like diphenyleneiodonium completely abolish mtDNA release. This confirms that NOX-derived ROS are strictly required [84]. Specific signals also induce mitochondrial NET release through pathways dependent on ROS and cell death. For instance, deacetylase Sirtuin 1 (SIRT1) opens the mitochondrial permeability transition pore to provide a release route [88]. The mitochondrial inner membrane protein optic atrophy 1 (OPA1) supports glycolysis to produce ATP. This energy reorganizes microtubules, providing structural tracks for mtDNA transport [89]. Furthermore, conditioned media from anaplastic thyroid cancer cells stimulate healthy neutrophils. This increases ROS production to drive mtDNA extrusion [90].

Collectively, neutrophils deploy complementary suicidal, vital, and mitochondrial pathways. Together, they form a multilayered and dynamic host defense system. While other modes likely exist, elucidating these core mechanisms remains imperative. This research deepens our understanding of innate immune complexity. It also offers targeted therapies for NET-mediated inflammation and autoimmunity.

### The journey of NETs in the CNS

Neutrophils are predominantly confined to the peripheral circulation by the BBB and are seldom found within the healthy CNS [91]. Under pathological conditions, however, they can breach the BBB, infiltrate the CNS parenchyma, and excessively form NETs under local microenvironment stimulation. Overproduction or impaired clearance of NETs can cause a series of diseases [92, 93]. Neurons and glial cells can sense NETs and mount a response through various mechanisms [94, 95].

#### Mechanisms of neutrophil infiltration into the brain

**Fig. 3:**
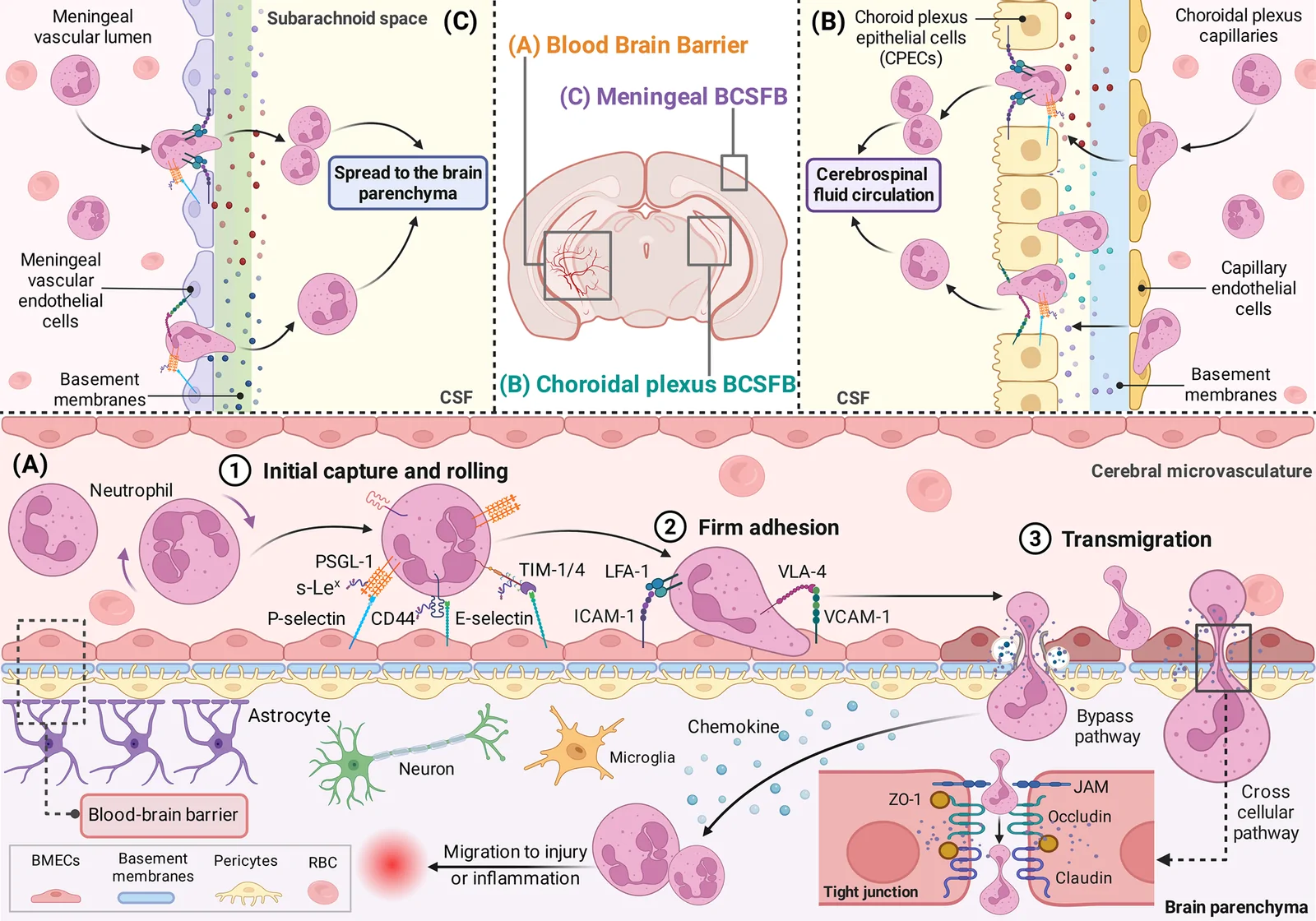
Neutrophil recruitment across brain barriers and NET formation. Neutrophil trafficking into the brain parenchyma via three brain barriers: (**A**) BBB, (**B**) The choroid plexus BCSFB, and (**C**) The meningeal BCSFB. Neutrophils undergo sequential processes at the BBB: initial capture/rolling (mediated by PSGL-1, selectins), firm adhesion (via LFA-1/ICAM-1, VLA-4/VCAM-1), and transmigration (paracellular via TJs or bypass pathways). At the choroid plexus BCSFB, neutrophils interact with epithelial cells and enter CSF circulation; at the meningeal BCSFB, they migrate from meningeal vessels to the brain parenchyma. Post-transmigration, neutrophils move to sites of injury

Neutrophil infiltration into the brain parenchyma is predominantly achieved through three distinct routes: the BBB, the blood-cerebrospinal fluid barrier (BCSFB), and the skull bone marrow-meningeal pathway [96–99]. This complex process is orchestrated by a repertoire of signaling molecules, regulating the generation and chemotaxis of inflammatory cues, the rolling, adhesion, and activation of the vascular endothelium, subsequent changes in BBB permeability and transmigration, and ultimate localization within cerebral tissue [100, 101] (Fig. 3).

Under physiological conditions, the BBB limits cellular infiltration through tightly sealed endothelial junctions. However, in pathological contexts such as brain injury, ischemia, or infection, damaged neurons and glial cells rapidly release pro-inflammatory cytokines, including IL-1β, TNF-α, CXCL1, and CXCL2 [96]. These cytokines form a concentration gradient in the circulation, prompting the migration of peripheral neutrophils toward the brain [96, 102, 103]. Concurrently, brain microvascular endothelial cells (BMECs) upregulate the expression of P-selectin and E-selectin, which interact with ligands on neutrophils, such as P-selectin glycoprotein ligand-1 (PSGL-1), CD44, T cell immunoglobulin and mucin-domain (TIM) [104, 105], and others to mediate initial capture and rolling along the endothelium [106, 107]. Subsequently, neutrophils establish firm adhesion through specific binding of integrins, leukocyte function-associated antigen-1 (LFA-1) and very late activation antigen-4 (VLA-4) to endothelial adhesion molecules intercellular adhesion molecule-1 (ICAM-1) and vascular cell adhesion molecule-1 (VCAM-1), respectively [108–110]. Once firmly adhered, neutrophils traverse the BBB via either paracellular or transcellular pathways. The paracellular pathway involves neutrophil passage through gaps between endothelial cells and the vascular wall, whereas the transcellular pathway relies on neutrophil movement through vesicular structures within endothelial cells and across the cell body [106, 111]. During transmigration, neutrophils secrete proteases that degrade endothelial tight junction (TJs) proteins, such as claudin-5, zonula occludens-1 (ZO-1), and occludin, thereby enhancing BBB permeability and facilitating further migration [112]. Finally, upon entering the brain, neutrophils migrate toward sites of injury or inflammation guided by chemotactic signals.

Crossing the BCSFB in the choroid plexus (ChP) and meninges is another key pathway [97, 113]. Upon inflammatory stimuli, chemokines (CXCL1, CXCL2/MIP-2, and CXCL8) from ChP epithelial cells, stromal fibroblasts, and meningeal immune cells diffuse from the ChP stroma (blood-proximal side) to the cerebrospinal fluid (CSF) side, forming a concentration gradient [114, 115]. Neutrophils, with high CXCR2 expression on their surface, sense this gradient and migrate toward CSF [114, 115]. Peripheral blood neutrophils first adhere to ChP capillary endothelial cells, then sequentially complete blood-ChP interstitial migration and interstitial-CSF migration [113]. Once in the interstitium, neutrophils bind to adhesion molecules on ChP epithelial cells, and inflammatory signals induce temporary gaps in epithelial TJs, enabling neutrophils to cross the epithelial cell layer into CSF [116]. In CSF, neutrophils diffuse to the ventricular system and subarachnoid space (SAS) via CSF circulation, thereby affecting periventricular or meningeal damaged areas [117]. In addition, they migrate to the brain parenchyma through the pia mater-brain parenchyma perivascular (Virchow-Robin) gap, supplementing BBB-infiltrated neutrophils and jointly participating in inflammation or pathogen clearance [118]. The migratory mechanism of meningeal BCSFB is similar to that of ChP-BCSFB. Neutrophils adhere to the endothelial lining of meningeal blood vessels and traverse the vascular wall directly into the CSF of the SAS, bypassing the epithelial cell layer. This pathway is more active in meningeal local inflammation, rapidly recruiting neutrophils to the meningeal surface and limiting infection spread to the brain parenchyma [113, 119].

Notably, the skull bone marrow-dura mater direct channel is a recently identified shortcut, with small vascular channels between them [120]. Neutrophils from skull and vertebral bone marrow can directly infiltrate the brain parenchyma, showing a distinct transcriptional profile from peripheral blood cells [121]. This suggests local hematopoietic reservoirs’ unique role in brain immune surveillance.

Collectively, among the three pathways, the BBB primarily mediates neutrophil infiltration into the brain parenchyma, the BCSFB facilitates neutrophil diffusion into the CSF, and the direct trans-skull migration pathway enables rapid, targeted recruitment of immune cells. These pathways rely on multi-level synergistic mechanisms, including inflammatory chemokine-driven chemotaxis, selective integrin-mediated adhesion, protease-induced disruption of TJs, and local bone marrow-derived neutrophil supply [122]. Together, they mediate neutrophil migration to the CNS, providing critical targets for neuroimmunology and cerebrovascular disease treatment [123].

#### The triggering signal of NETosis in the brain

**Fig. 4:**
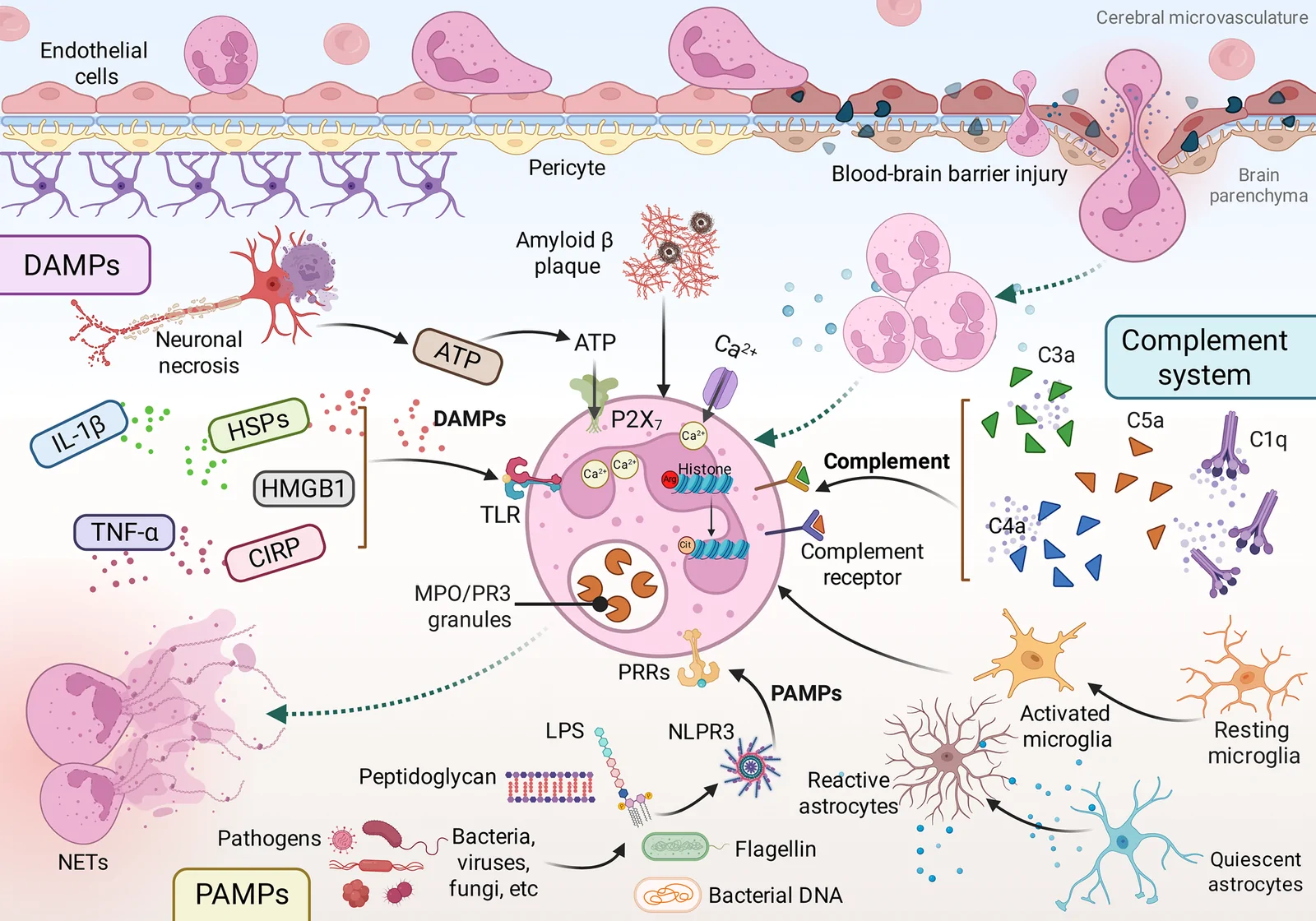
The triggering signal of NETosis in the brain. The initiation of NETosis involves DAMPs, PAMPs, and the complement system. Upon exposure to DAMPs (ATP, HMGB, Aβ, and CIRP) and PAMPs (LPS and bacterial DNA), neutrophils undergo activation via TLR/PRR signaling. Concurrently, interactions with the complement system drive neutrophils to release granular components (MPO/PR3) and NETs. Additionally, neutrophil infiltration into the brain parenchyma triggers the activation of microglia and astrocytes, which further exacerbates NET formation

After infiltrating brain tissue, neutrophils undergo NETosis and form NETs in response to various signals within the brain microenvironment [124, 125]. NETosis is not triggered by a single signal but is driven by damage-associated molecular patterns (DAMPs), pathogen-associated molecular patterns (PAMPs), and the complement system [126, 127] (Fig. 4).

DAMP-mediated signaling is a key trigger for NETosis in non-infectious CNS injuries. For example, histone fragments produced by glial cells in neuroinflammation, cold-induced RNA binding protein (CIRP), high mobility group protein B1 (HMGB1), and ATP released by neuronal necrosis following cerebral ischemia [128, 129]. ATP activates the P2×7 receptor on neutrophils and causes rapid intracellular Ca^2+^ influx [128]. HMGB1 and CIRP activate MyD88-NF-κB and p38 MAPK signaling by binding to TLR4 on neutrophil surfaces, further amplifying NETosis-related signals [130–133].

PAMPs are typically found in infectious CNS disorders [134]. Treatment with LPS enhances the brain’s neutrophil activation, which raises NE and H3cit, both of which indicate an increase in NET release [135, 136]. Additionally, LPS increases NLR Family Pyrin Domain Containing 3 (NLRP3). While not affecting neutrophil infiltration, NLRP3 increases DNA leakage and neutrophil mortality, worsens the development of NETs, and causes BBB degradation [137]. Existing studies have demonstrated that PAMPs produced from viruses can successfully cause peripheral neutrophil NETosis. For instance, HIV-1 appears to regulate NETosis in a context-dependent manner. Viral nucleic acids can be recognized by TLR7/8 on neutrophils and promote ROS-dependent NET formation, whereas HIV-1-induced IL-10 may suppress NET release and facilitate immune evasion [138]. Respiratory syncytial virus particles and fusion proteins can function as PAMPs by binding to neutrophil TLR4 in a concentration-dependent manner, triggering ROS produced from NOX2, encouraging activation of ERK and p38 MAPK, and eventually causing the release of NETs [139]. Through neutrophil TLR7, the viral nucleic acid of the chikungunya virus may trigger NETosis, and there is a positive correlation between the viral load and the degree of NETosis in acute chikungunya virus-infected humans [140]. However, the mechanism by which PAMPs of this kind of virus cause neutrophil NETosis in the brain environment is not yet well understood.

Furthermore, complement components may enter as a result of BBB degradation [141, 142]. Excessive deposition of activated complement molecules, such as C1q, can inhibit DNase activity, thereby promoting NET accumulation [143]. Other complement system molecules, including CR1, recognize beta-amyloids (Aβ) plaques opsonized by C3b fragments and preferentially induce NET release [142]. C5a recruits peripheral neutrophils to Aβ plaques by binding to C5aR1 receptors on the neutrophil surface, further facilitating NET extrusion [142]. Concurrently, C5a can activate G protein-coupled signaling via neutrophil C5aR1, accelerating Ca^2+^ influx and ROS generation, which indirectly provides synergistic stimulation for NETosis [144, 145]. Moreover, activation of microglia and astrocytes further enhances NET release [146, 147]. Notably, Ca^2+^ influx induced by cerebral ischemia promotes NET formation [128], while high concentrations of glutamate in brain tissue can augment Ca^2+^ influx through NMDA receptors [148]. These findings suggest that specific metabolic signals within the CNS microenvironment may also participate in regulating NETosis. Subsequently, the brain translates the physical presence of NETs into robust chemical inflammatory signals via its innate immune cells, thereby perceiving this “foreign threat”. Although this perception mechanism is protective in nature, aiming to clear threats, its dysregulation can drive chronic neuroinflammation and secondary brain damage, highlighting the critical role of NETs as a key hub in neuroimmunopathology.

### Interpretation of evidence for NETs in CNS diseases

The crux of investigating NETs in CNS diseases lies not merely in identifying elevated signals, but in determining whether these signals represent general neutrophil activation, extracellular chromatin release, the formation of structures resembling NETs, or a level of evidence robust enough to implicate NETs as active pathological drivers [149]. NETs were originally defined as reticular networks composed of an extracellular DNA backbone bound to neutrophil granular proteins, such as MPO and NE. Therefore, reliable identification typically necessitates the spatial colocalization of extracellular DNA with proteins derived from neutrophils, rather than relying on any single biomarker [3, 150]. While elevated levels of cell-free DNA (cfDNA), MPO, NE, H3cit, or complexes comprising MPO and DNA may indicate processes related to NETs, these metrics alone fail to establish NETs as causal mediators of disease progression [151, 152]. Robust evidence supporting the pathological significance of NETs must demonstrate their localization within the meninges, the brain parenchyma, or blood vessels associated with lesions. It is equally crucial to establish a temporal correlation between NET accumulation and tissue damage, alongside evidence of functional reversal achieved through NET degradation or the inhibition of their formation [51, 153, 154].

The specificity of NET biomarkers warrants cautious interpretation. Because cfDNA can originate from apoptosis, necrosis, tissue injury, and various other forms of extracellular DNA release, it does not serve as a specific product of NETosis [152, 155]. Although MPO and NE are critical indicators of neutrophil activation and degranulation, their release during neutrophil responses independent of NETosis precludes their use as standalone evidence for NET formation [156]. H3cit remains a widely utilized biomarker for NETs; however, its detection and quantification are heavily influenced by antibody selection, sample processing, assay platforms, and its binding status to DNA [157]. Compared to individual markers, detecting complexes of MPO and DNA or H3cit and DNA more accurately reflects the composite architecture of NETs. Nevertheless, circulating complexes still present limitations regarding specificity and the interpretation of their origins, as they cannot directly differentiate whether the NETs emanate from the peripheral blood, cerebral vasculature, meningeal spaces, or parenchymal lesions [158].

Evidence for NETs in CNS diseases must be evaluated comprehensively by integrating sample origin, spatial localization, and functional interventions, rather than inferring causality solely from elevated peripheral blood biomarkers. Clinical investigations need to explicitly state whether signals related to NETs are derived from serum, plasma, CSF, thrombectomy specimens, postoperative brain tissue, or postmortem brain tissue, given that different sample types carry distinct anatomical origins and pathological implications [159, 160]. Animal studies must similarly distinguish the global effects of neutrophils from the specific contributions of NETs. For example, while depleting neutrophils via antibodies directed against Ly6G can alleviate microvascular obstruction or inflammatory damage, this observation alone does not confirm that NETs are the primary pathogenic factor [161]. DNase I should be strictly classified as a strategy to degrade the extracellular DNA backbone of already formed NETs, rather than as an agent that inhibits their initial formation [162]. Conversely, inhibitors targeting PAD4 primarily curtail NET formation by restricting histone citrullination and chromatin decondensation [163].

The strength of evidence implicating NETs varies considerably across different CNS pathologies. Current evidence is particularly robust for ischemic stroke, as NETs are directly observable in the brain tissue of affected patients, and the NET content within thrombi correlates with both stroke outcomes and the response to tissue plasminogen activator (tPA) thrombolysis [164]. Animal models provide further substantiation, demonstrating that NETs contribute to impaired vascular remodeling, BBB disruption, microvascular perfusion deficits, and neurological impairment [12]. Moreover, interventions such as NET degradation, PAD4 inhibition, or the administration of NET inhibitory peptides can attenuate brain injury in experimental stroke models, thereby strengthening the causal link between NETs and stroke pathology [51, 92]. Traumatic brain injury (TBI) also benefits from strong experimental support, characterized by elevated circulating NETs and reduced DNase I activity in clinical cohorts. In parallel, experimental models show that NET formation correlates with cerebral hypoperfusion, tissue hypoxia, and neurological deficits [153]. Animal studies additionally indicate that NETs drive coagulation abnormalities and endothelial damage, while treatment with DNase I mitigates these coagulopathies [165]. However, the clinical evidence for causality in TBI remains less definitive than in stroke. Research on subarachnoid hemorrhage (SAH) suggests that NETs participate in cerebrovascular occlusion, cortical hypoperfusion, and delayed cerebral ischemia, with strategies targeting neutrophils or NETosis showing promise in improving experimental SAH outcomes [154]. Clinical SAH studies have similarly identified associations between biomarkers related to NETs and the risk of delayed cerebral ischemia; nevertheless, their predictive value, optimal detection windows, and ideal intervention timing require further clinical validation [166]. In the context of chronic conditions such as Alzheimer’s disease (AD) and multiple sclerosis (MS), NETs are more accurately conceptualized as potential amplifiers of vascular inflammation, barrier dysfunction, protein deposition, or autoimmune responses, rather than definitively established primary etiologies [110, 167].

### NET-mediated neurological disorders

In the following disease-focused sections, we do not treat the presence of NET markers as equivalent to pathogenic causality. Instead, we evaluate NET evidence according to clinical association, anatomical localization, mechanistic causality, and therapeutic reversibility. This distinction is important because NET markers may reflect systemic inflammation, neutrophil activation, or secondary tissue injury rather than a primary disease-driving mechanism. Therefore, each section emphasizes not only whether NETs are present, but also how strongly current evidence supports their contribution to disease pathophysiology.

#### NETs in stroke

Stroke is an acute cerebrovascular disorder in which vascular occlusion or rupture rapidly disrupts cerebral perfusion and triggers neurovascular injury [168]. In stroke patients, circulating NET-related markers and NET-rich thrombi have been associated with thrombus burden, impaired reperfusion, thrombolytic resistance, and poor clinical outcome, supporting their potential value as vascular biomarkers and thromboinflammatory contributors in stroke [15, 164, 169]. NETs can appear within 6 hours after stroke and peak at 24–48 hours, with most NETs located in the vascular compartment and a smaller proportion in murine brain parenchyma [170]. Injured endothelial cells upregulate inflammatory cytokines, chemokines, and adhesion molecules, thereby promoting immune cell recruitment, local NET deposition, fibrinogen accumulation, and vascular leakage [171]. In acute ischemic stroke (AIS), CXCL2^+^ neutrophils in the brain border zone display enhanced NET-forming capacity, and H3cit^+^ neutrophil accumulation further impairs vascular reperfusion, neovascularization, capillary perfusion, BBB repair, and neurological recovery [12, 172].

Several upstream signals converge on NET formation after stroke. Platelet-derived HMGB1 induces H3cit and MPO-DNA formation in neutrophils, and activated platelets can further release microvesicles and HMGB1 to promote autophagy-related NETosis involving LC3B and Beclin-1 [16, 51, 53, 173]. HMGB1 also activates platelets through TLR4-dependent signaling, strengthening platelet-neutrophil-endothelial interactions and promoting the capture of procoagulant factors and red blood cells [174, 175]. Other DAMP- and metabolism-related pathways may reinforce this response. ATP released from damaged neurons and glia activates P2×7 receptors on neutrophils, triggering Ca^2+^ influx, protein kinase C alpha (PKCα) activation, NOX2-dependent ROS generation, PAD4 activation, and chromatin release [128, 176]. Notably, PKCα also mediates lamin B phosphorylation, resulting in nuclear membrane disruption and chromatin release, which facilitates NET assembly [177]. Neuronal Sp1 can promote CIRP release, which induces NET formation through TLR4/p38 signaling and aggravates endothelial barrier disruption [133]. Pyruvate kinase M2 (PKM2) overactivation enhances NETosis and upregulates MPO, NE, hypoxia-inducible factor 1 alpha (HIF-1α), and NF-κB p65, whereas PKM2 deletion reduces peripheral neutrophil inflammation and NET formation after cerebral ischemia-reperfusion [52, 178]. In contrast, lipoxin A4 limits brain injury by downregulating methyltransferase-like 3 (METTL3) and suppressing NET-associated proteases and neutrophil ferroptosis, while phenylacetylglutamine may promote NET formation and systemic inflammation in diabetes-associated ischemic stroke [179]. In diabetes-associated ischemic stroke, stroke- and type 2 diabetes-induced gut dysbiosis increases circulating phenylacetylglutamine, a gut microbiota-derived metabolite. Phenylacetylglutamine promotes neutrophil NET formation, systemic inflammation, and oxidative stress, thereby aggravating ischemic brain injury [180].

Pathologically, NETs amplify stroke injury through vascular toxicity, inflammatory signaling, and immunothrombosis. NET-associated cathepsin G, NE, and MPO damage cerebrovascular endothelial cells, disrupt tight junction proteins such as ZO-1, occludin, and claudin-5, and impair adherens junctions, thereby increasing BBB permeability and the risk of hemorrhagic transformation or neurological deterioration [12, 181, 182]. Extracellular DNA from NETs can activate the cGAS-STING pathway, inducing TBK1 and IRF3 phosphorylation and increasing IFN-β, TNF-α, and IL-6 production, which further aggravates neuroinflammation and vascular injury [12, 181, 183]. NETs also promote platelet and endothelial activation, increasing von Willebrand factor (VWF) and plasminogen activator inhibitor-1 (PAI-1) release and reinforcing a procoagulant phenotype [184]. In a murine stroke model, infiltrating neutrophil-derived NETs activate the Absent in Melanoma 2 (AIM2) inflammasome in microglia, triggering pyroptosis and exacerbating ischemic brain injury [95, 185]. Consistently, elevated plasma NET levels together with reduced endogenous DNase activity are associated with accelerated stroke pathology and worse prognosis [51, 186].

The interaction between NETs and thrombolytic therapy further supports their relevance to stroke outcome. Although tPA remains a major treatment for AIS, it can increase vascular permeability and promote neutrophil adhesion and migration into ischemic tissue [187–189]. These neutrophils interact with low-density lipoprotein receptor-related protein 1 (LRP-1), upregulate PAD4, and promote H3 citrullination and NET formation, which may increase thromboinflammatory injury and reduce thrombolytic efficacy [47, 181, 190]. Interventions targeting NET formation or promoting NET degradation, including platelet HMGB1 depletion, DNase I-mediated degradation of already formed NETs, sivelestat, activated protein C (APC), and neonatal NET inhibitory peptide, reduce circulating and cerebral NET burden, limit neuronal apoptosis, and attenuate neuroinflammation in experimental models [51, 165, 191, 192].

**Fig. 5:**
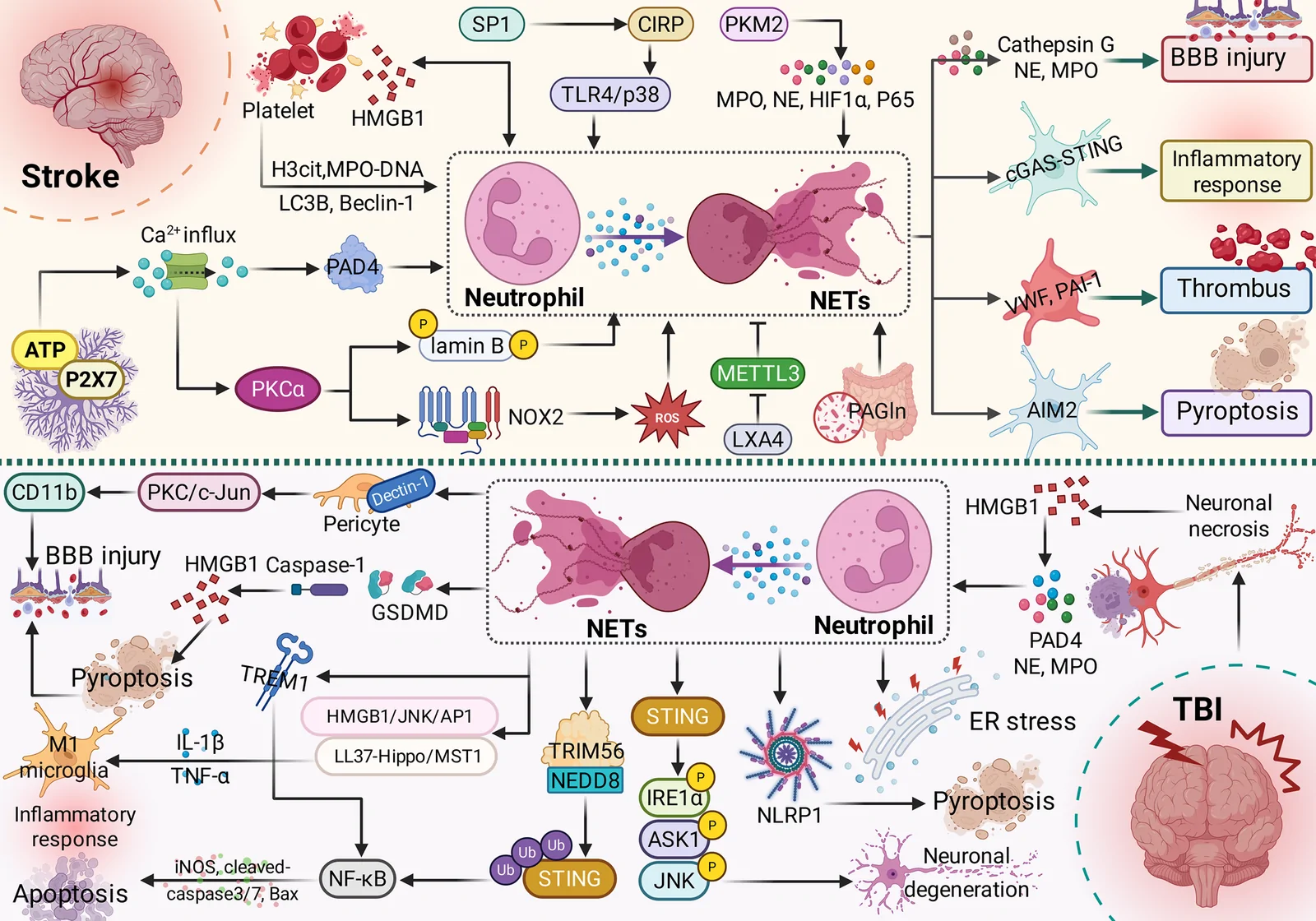
NET-driven pathological cascades in stroke and TBI. In stroke and TBI, stimuli including Ca^2+^ influx, HMGB1, and ATP-P2 × 7 signaling induce NET formation via pathways involving PAD4, NOX2, and METTL3. Released NETs mediate multiple pathological processes: BBB injury (via cathepsin G and NE), inflammatory responses (the cGAS-STING axis), thrombosis (VWF and PAI-1), and pyroptosis (the AIM2 pathway). Additionally, NETs interact with pericytes, microglia, and neurons to promote neuroinflammation, ER stress, and neuronal damage, while feedback loops (HMGB1 and STING) amplify this pathogenic cascade

Taken together, stroke currently represents one of the disease contexts with the most compelling evidence for the pathogenic role of NETs. Induced through multiple molecular pathways, NETs exacerbate cerebral injury by releasing cytotoxic components, activating inflammatory cascades, and promoting coagulation abnormalities (Fig. 5). The chain of evidence encompasses elevated acute phase markers of NETs, their spatial localization within thrombi and the vasculature of ischemic regions, and their immunothrombotic interactions with VWF, plasminogen activator inhibitor 1, fibrin, and platelets. This is further corroborated by the protective effects observed in experimental models following interventions such as DNase I administration, PAD4 inhibition, NE inhibition, and the application of NET inhibitory peptides. Consequently, within the context of stroke, NETs likely function as pathological amplifiers of immunothrombosis, reperfusion failure, and blood-brain barrier disruption, rather than merely serving as accompanying markers of inflammation. Nevertheless, current clinical evidence is predominantly derived from observational studies, and biomarkers for NETs remain susceptible to confounding factors, including stroke severity, concurrent infections, systemic inflammation, and therapeutic interventions. Clarifying the causal weight of NETs in human stroke necessitates simultaneous dynamic analyses of patient thrombi, CSF, and peripheral blood, combined with spatial imaging and interventions strictly targeted at NETs.

#### NETs in traumatic brain injury

TBI is caused by external mechanical force and involves both primary mechanical damage and delayed secondary injury. Secondary injury is driven by BBB disruption, vascular dysfunction, edema, coagulation abnormalities, neuroinflammation, and progressive neuronal death [153]. NETs are present in the serum and brain tissue of TBI patients and animal models, and are associated with poor disease outcomes [193, 194]. HMGB1, released from necrotic neurons following TBI, activates TLR4 on neutrophils to promote PAD4‑dependent NET formation and upregulate the expression of MPO and NE [153, 195, 196]. Meanwhile, reduced serum DNase I activity has been observed in patients with severe TBI and correlates with increased circulating NETs [153]. Experimental data further suggest that TLR4 activation may contribute to this reduction, thereby promoting NET accumulation by both enhancing NET formation and limiting NET degradation [153]. This dual process, increased NET generation together with insufficient NET clearance, may be a defining feature of NET pathology in TBI.

NETs amplify vascular injury through coagulation, endothelial damage, and BBB disruption. They promote hypercoagulability by exposing phosphatidylserine and tissue factor, while also reducing endothelial protective molecules such as syndecan-1, thrombomodulin, and VWF [165, 197]. VWF may facilitate NET formation by supporting platelet adhesion, neutrophil recruitment, and platelet-neutrophil interactions at injured vessels. Consistently, VWF deficiency markedly reduces brain NET accumulation, suggesting that VWF functions not only as a coagulation factor but also as an adhesive scaffold for NET-associated thromboinflammation after brain injury [170]. NET-associated histones can be recognized by Dectin-1 on peri-brain cells, activating PKC/c-Jun signaling and inducing CD11b expression, which enhances leukocyte infiltration and disrupts BBB integrity [198]. NETs also activate NF-κB signaling and promote triggering receptor expressed on myeloid cells 1 (TREM1) accumulation and interaction with NF-κB, thereby amplifying endothelial inflammation and vascular spasm [199–201]. In parallel, NETs can trigger caspase-1/GSDMD-mediated endothelial pyroptosis, followed by injurin1 (NINJ1)-dependent membrane rupture and HMGB1 release, which further feeds back to promote NET formation and BBB injury [201, 202]. These findings suggest that TREM1, TLR4, PAD4, and NINJ1 may represent potential intervention points in NET-driven vascular dysfunction after TBI.

Beyond vascular injury, NETs contribute to neuroinflammation and neuronal damage. NET accumulation increases peripheral immune cell infiltration, BBB disruption, brain edema, and cerebral hypoperfusion [203, 204]. NETs also promote pro-inflammatory microglial activation through HMGB1/JNK/AP1 and LL37-Hippo/MST1 pathways, increasing IL-1β and TNF-α release and enhancing sympathetic neuronal overactivity [205, 206]. Several downstream neuronal injury pathways have been identified. NETs can enhance neddylation and activate the NEDD8–TRIM56-STING-NF-κB axis, thereby increasing neuroinflammation and neuronal apoptosis [20, 207]. They also activate STING-dependent IRE1α/ASK1/JNK signaling in neurons and microglia, leading to neuronal degeneration and apoptosis [203]. In addition, neutrophils in the TBI brain injury area and peripheral blood increase the expression of MPO, PAD4, and H3cit, and subsequently, NETs promote the expression of P-IRE1α, NLRP1, ASC, caspase‑1 p20, N‑GSDMD, IL‑18, and IL‑1β in the cerebral cortex, further exacerbating neuronal pyroptosis [208]. NET recognition by TLR9 may further induce ER stress and apoptosis-related molecules, including p-IRE1α, XBP-1s, GRP78, CHOP, cleaved caspase-3, and cleaved PARP-1, thereby aggravating TBI-related neuronal injury [209–211].

In summary, the strength of evidence implicating NETs in TBI ranges from moderate to relatively robust. A defining characteristic within this context is the concurrent increase in NET formation and the decline in DNase I activity, suggesting that the accumulation of NETs likely stems from a combination of augmented generation and impaired clearance mechanisms. Experimental models substantiate the mechanistic links between NETs and the disruption of the blood-brain barrier, endothelial damage, microcirculatory dysfunction, microglial activation, ER stress, inflammation associated with the STING pathway, and subsequent neuronal death (Fig. 5). Nevertheless, TBI exhibits profound clinical heterogeneity. Confounding variables such as hemorrhage, systemic trauma, inherent infection risks, and surgical interventions can all independently influence neutrophil activation and the levels of biomarkers related to NETs. Consequently, within the paradigm of TBI, NETs are most accurately characterized as pathological amplifiers of secondary injury cascades, rather than as universally established primary etiologies.

#### NETs in subarachnoid hemorrhage

SAH is a severe hemorrhagic stroke most commonly caused by rupture of an intracranial aneurysm. Its poor outcome is driven not only by the initial bleeding event but also by early brain injury, neuroinflammation, microthrombosis, cortical hypoperfusion, and delayed cerebral ischemia [212, 213]. In a mouse model of SAH, neutrophils undergo extensive cerebral infiltration and form intravascular NETs within 24 hours, coinciding with the peak expression of the NET-associated marker H3cit [154]. H3cit co-localizes with fibrinogen-positive microthrombi, providing a scaffold for microthrombus formation. This leads to microvascular occlusion in the brain and causes cortical hypoperfusion [214]. Depleting neutrophils with anti-Ly6G antibodies reduces microthrombus formation [215]. Over time, NETs progressively accumulate in the ipsilateral and contralateral brain parenchyma, spreading to the basal, cortical, and periventricular regions within 14 days [216]. The continuous accumulation of NETs promotes delayed cerebral ischemia, which manifests as a reduction in cerebral blood flow, cerebral infarction, and delayed neurological deficits [154]. This is because PAD4 inhibitors block NET generation at the source, intervene in the pathological upstream process, and reduce the initial formation and accumulation of NETs. In contrast, DNase I only degrades pre-formed NETs and has limited efficacy in preventing sustained NET generation [217, 218]. Interestingly, neutrophils derived from skull bone marrow have a higher propensity for NETosis, while neutrophils from femoral bone marrow exhibit a reduced tendency for NETosis [98, 154]. This difference is attributed to the heterogeneity of neutrophil sources. Neutrophils from femoral bone marrow must be recruited to the brain via peripheral circulation, where they are influenced by circulating regulatory factors or weak activation signals, leading to a reduced tendency for NET formation. Moreover, after SAH, peripheral bone marrow neutrophil activation may be inhibited, further weakening their ability to form NETs.

**Fig. 6:**
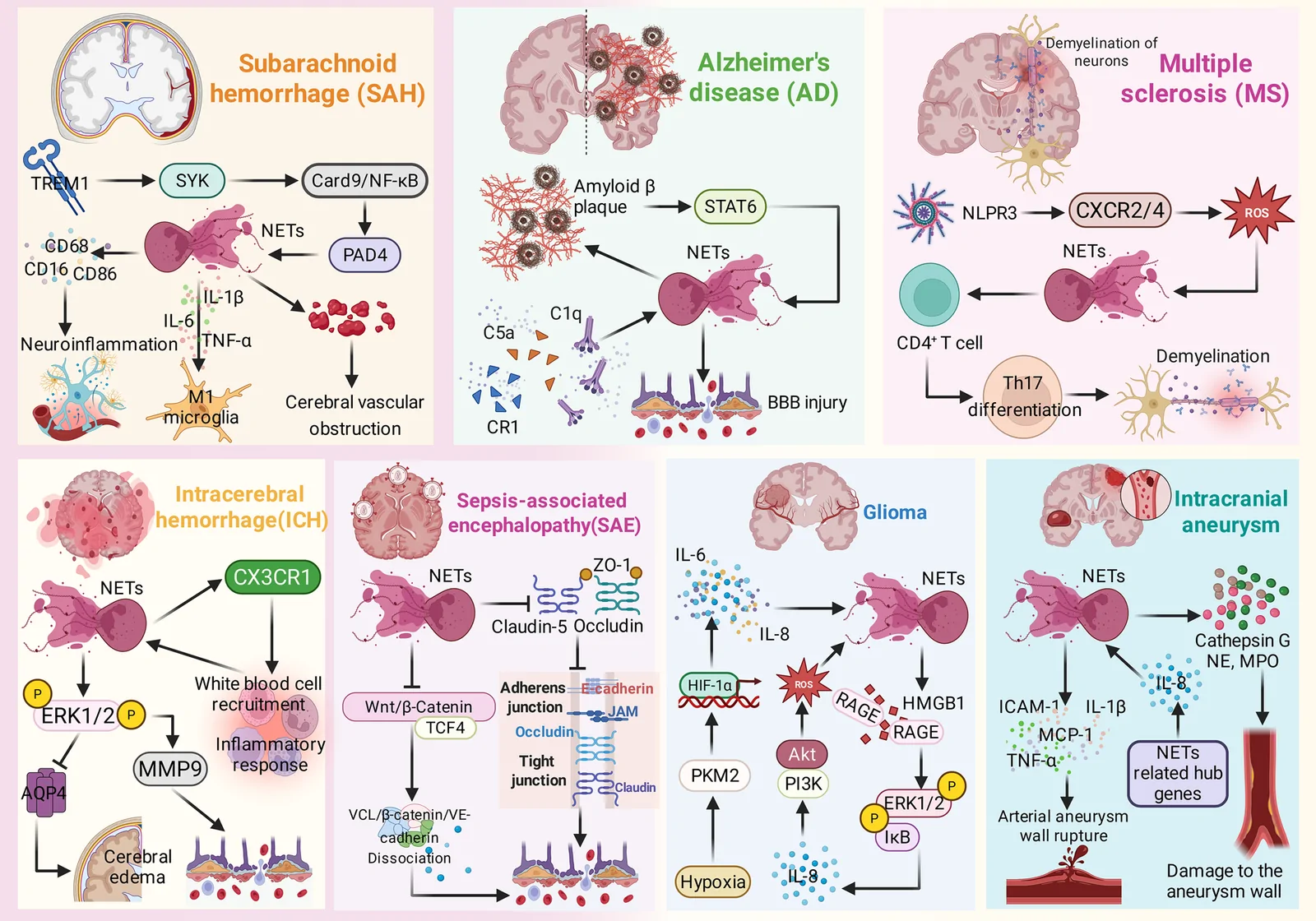
NET-mediated pathogenesis of diverse neurological disorders. In SAH, the TREM1/SYK pathway induces NET formation, driving neuroinflammation, cerebrovascular obstruction, and edema. In AD, NETs interact with Aβ plaques and complement components to trigger BBB injury. In MS, NETs promote Th17 cell differentiation and neuronal demyelination via the NLRP3/CXCR2 axis. In ICH, NETs mediate inflammatory responses and cerebral edema through the CX3CR1/ERK pathway. In SAE, NETs disrupt BBB TJs via Wnt/β-catenin signaling. In gliomas, NETs exacerbate hypoxia-driven inflammation through the HMGB1/ERK pathway. In intracranial aneurysms, NETs contribute to vascular wall damage through ICAM-1, MCP-1, cathepsin G, and MPO

Neuroinflammation plays a crucial role in the pathogenesis of SAH, and NETs are involved in this process. Mechanistically, after SAH, TREM1 activates SYK, thereby increasing Card9/NF-κB and PAD4/NETs signaling [219]. However, the formation of NET markers can be blocked by the TREM1 inhibitory peptide LP17, which downregulates the expression of pro-inflammatory microglial markers, such as CD68 and CD86, while reducing the levels of neuroinflammatory cytokines, including IL-6, IL-1β, and TNF-α [219, 220]. Similarly, NETs significantly exacerbate microglial inflammation and increase the levels of TNF-α, IL-1β, and IL-6 [221]. In contrast, inhibiting NET formation with GSK484, a selective PAD4 inhibitor, exhibits anti-inflammatory effects by reducing these pro-inflammatory factors [221] (Fig. 6).

In brief, the evidence implicating NETs in SAH is relatively robust. NETs emerge early within the cerebral vasculature and correlate with elevated levels of H3cit, microthrombus formation, cortical hypoperfusion, and delayed cerebral ischemia. Interventions such as PAD4 inhibition, DNase I administration, or neutrophil depletion effectively mitigate the microthrombus burden, reductions in cerebral blood flow, and neuroinflammation. These findings indicate that NETs likely function as critical amplifiers of microcirculatory dysfunction and secondary brain injury following SAH. However, current evidence remains predominantly derived from animal models and static tissue biomarkers. Consequently, longitudinal clinical profiling and the definitive establishment of causal relationships require further robust validation.

#### NETs in Alzheimer’s disease

AD is a chronic neurodegenerative disorder characterized by progressive cognitive decline, Aβ deposition, tau pathology, synaptic dysfunction, and neuroinflammation [222]. Studies have shown that the pathogenesis of AD is closely associated with innate immune cells, particularly neutrophils [223, 224]. Once in the brain, neutrophils release NETs and ROS in both the blood vessels and parenchyma, contributing to BBB disruption [225, 226]. Clinical studies have shown a significant increase in the expression of NETs and key NET-associated genes (CCL2 and TLR2) in the peripheral blood of AD patients, and NETosis has been observed in the AD vasculature [17, 227]. However, neutrophils are primarily found in the blood vessels, with fewer detected in the brain parenchyma of AD patients and animal models. It is important to note that the discrepancy in neutrophil distribution may be influenced by sample differences, as the timing of neutrophil death and perfusion could affect their presence in the vascular system [17].

In AD, neutrophils aggregate and release MPO, which damages brain endothelial cells through the production of hypochlorous acid (HOCl) and hypothiocyanous acid, thereby disrupting the integrity of the BBB [228, 229]. Mechanistically, Aβ deposited in cerebral blood vessels activates STAT6 signaling in neutrophils, triggering a shift from programmed cell death to NETosis [230]. The released NETs not only directly damage the vascular endothelium but also serve as scaffolds for further Aβ deposition. Moreover, chronic stress increases norepinephrine circulation, which enhances neutrophil chemotaxis to the cerebral vasculature and exacerbates NET formation by activating STAT6, thereby promoting cerebral amyloid angiopathy [230]. In an AD transgenic mouse model, neutrophils infiltrate the Aβ deposition areas, releasing NETs and IL-17 [110]. Inhibition of LFA-1 prevents neutrophil extravasation and NET release, reducing AD-like lesions and improving cognitive function [110]. Furthermore, pharmacological interventions with Cl-amidine and Disulfiram can disrupt NET formation, restore BBB integrity, and alleviate inflammation [231]. It is also noteworthy that components of the complement system are implicated in AD pathogenesis [232]. C5a, C1q, and CR1 are involved in recruiting granulocytes and promoting NET release, which exacerbates AD [142] (Fig. 6). Therefore, regulating the complement system to inhibit NET formation may be a promising strategy for delaying disease progression. However, the specific relationship between the complement system and NETs remains incompletely understood.

Compared with stroke and TBI, the causal evidence linking NETs to AD progression remains less mature. Current studies support vascular and perivascular neutrophil activation, NET-associated BBB injury, and potential interactions with Aβ pathology, but it remains uncertain whether NETs are primary drivers of neurodegeneration or secondary amplifiers of vascular inflammation and protein deposition. Therefore, NETs in AD should currently be interpreted as plausible contributors rather than established causal mediators.

#### NETs in multiple sclerosis

MS is an autoimmune demyelinating disease of the CNS in which peripheral immune activation, CNS immune cell infiltration, and myelin-directed inflammation jointly impair neural conduction [233]. Human evidence supports a systemic neutrophil-activation state in MS, as neutrophils from patients are more numerous, show reduced apoptosis, increased TLR2, fMLP receptor, IL-8 receptor, and CD43 expression, enhanced degranulation and oxidative burst, and are accompanied by higher serum levels of NETs [234]. Serum MPO is also increased in MS patients and is associated with clinical severity [235]. In addition, systemic neutrophil-related factors, including neutrophil elastase, CXCL1, and CXCL5, correlate with MS lesion burden and clinical disability, further linking neutrophil activation to structural and functional disease severity [236].

Experimental evidence further supports a role for NETs within the CNS compartment in MS-like pathology. In the experimental autoimmune encephalomyelitis (EAE) mouse model, CNS-recruited neutrophils produce cathelicidin-related AMP (CRAMP)-rich NETs during the early disease stage, and this response is required for EAE initiation [237]. In another EAE model, activation of NLRP3 upregulates CXCR2 and CXCR4 on infiltrating neutrophils in the brain, thereby promoting ROS-dependent NET formation accompanied by neutrophil death [137, 238]. This NLRP3-related NETosis appears to be PAD4-independent in this setting, suggesting that NET formation in MS-like CNS inflammation may depend on disease stage, tissue compartment, and local inflammatory cues rather than a single canonical pathway [239]. NETs generated in this process can further regulate NLRP3 through feedback loops, directly stimulate CD4^+^ T-cell activation, promote Th17 differentiation, and facilitate antigen presentation, thereby recruiting additional Th1 and Th17 cells into the CNS and exacerbating inflammation and demyelination [167, 239, 240].

PAD-dependent citrullination pathways may also participate in MS pathogenesis. Whole-transcriptome analysis showed that PAD2 and PAD4 are upregulated in relapsing-remitting MS but downregulated in secondary progressive MS, indicating that PAD-related inflammatory and citrullination signatures may differ across MS subtypes [241]. Therapeutically, non-covalent PAD inhibitors targeting PAD2 and PAD4 improved clinical outcomes in EAE and showed efficacy in demyelination models, supporting PAD inhibition as a potential strategy for limiting autoimmune demyelinating injury [242]. However, because PAD enzymes regulate broader protein citrullination beyond NETosis, the protective effects of PAD inhibition should not be interpreted as exclusively NET-dependent. Furthermore, FTY720, an immunosuppressant used in MS treatment, can induce NET formation in vitro [243]. Notably, FTY720-induced NET formation does not require NOX activity or PAD4 but depends on p38 and AKT signaling pathways [244]. Nevertheless, these observations currently reflect in vitro mechanisms, and whether FTY720-associated NET formation influences MS progression in vivo remains to be clarified (Fig. 6).

In general, the evidence supports an emerging role of NETs in CNS autoimmunity, particularly in neutrophil activation, Th17-related inflammation, and demyelinating injury. However, many human data still rely on circulating neutrophil or NET-associated markers rather than direct intrathecal or lesion-based NET visualization. Therefore, NETs should be considered potential amplifiers of inflammatory demyelination, while their stage-specific and compartment-specific roles require further validation.

#### NETs in intracerebral hemorrhage

Intracerebral hemorrhage (ICH) results from rupture of cerebral blood vessels and causes primary hematoma formation followed by secondary injury, including hematoma expansion, perihematomal edema, neuroinflammation, and neuronal death [245]. High levels of NETs have been detected in both plasma and hematoma samples from patients with ICH [246]. Neutrophils and NETs are also present around dense fibrin in cerebral hematomas of patients who died from spontaneous ICH [247]. Following intraventricular hemorrhage, neutrophils infiltrate the meningeal lymphatic system and form NETs [248, 249], in addition to accumulating and forming NETs in the perihematoma edema area [250]. These NETs upregulate the expression of CX3CR1 on the surface of meningeal lymphatic endothelial cells, promoting leukocyte recruitment and adhesion, which exacerbates local inflammation and further induces NET formation [251]. Concurrently, NETs disrupt the morphology of meningeal lymphatic endothelial cells, reduce the expression of lymphangiogenic markers (PROX1, VEGFC, VE-cadherin, and LYVE-1), increase cell apoptosis, and impair CSF transport function in the meningeal lymphatic system [251]. However, degradation of NETs or knockdown of CX3CR1 can restore the drainage ability of the meningeal lymphatic system, alleviating hydrocephalus and neurological dysfunction following ICH [251]. Another mechanism involves the upregulation of matrix metalloproteinase 9 (MMP-9) and the reduction of Aquaporin-4 (AQP4) levels through ERK1/2 phosphorylation [250]. MMP-9 further degrades TJs proteins (ZO-1 and occludin), compromising BBB integrity, while reduced AQP4 levels hinder cerebral water balance regulation, exacerbating brain edema after ICH surgery [250]. These findings highlight the crucial role of NETs in ICH pathology and open new avenues for targeted therapies in treating ICH-related injuries [252] (Fig. 6).

In ICH and intraventricular hemorrhage, NETs are increasingly linked to hematoma-associated inflammation, meningeal lymphatic dysfunction, BBB injury, and edema formation. Nevertheless, the evidence is still largely model-dependent, and it remains unclear whether NETs are initiating mediators of injury or downstream amplifiers of hemorrhage-induced inflammation. Future work should define the temporal relationship among hematoma formation, neutrophil recruitment, NETs deposition, and lymphatic drainage failure.

#### NETs in sepsis-associated encephalopathy

Sepsis-associated encephalopathy (SAE) is a diffuse cerebral dysfunction caused by systemic infection without direct CNS invasion, driven by inflammation, microvascular dysfunction, and BBB disruption [253]. Against this background, excessive activation of NETs exacerbates inflammation, disrupts the integrity of the BBB, and weakens endothelial cell junctions [254]. NETs reduce the expression of TJs proteins such as ZO-1, occludin, and claudin-5, and promote the dissociation of VCL/β-catenin/VE-cadherin complexes by inhibiting the Wnt/β-catenin/TCF4 signaling pathway [254]. This ultimately compromises BBB integrity. Notably, the protective effect of inhibiting NET formation extends beyond TJs and includes the remodeling of AJs and cellular structures. Additionally, PD-L1 interacts with p-Y705–STAT3 in neutrophils to form a complex, which translocates to the nucleus and promotes GSDMD mRNA transcription [255]. Following activation of caspase-4/5/11, GSDMD forms pores in both the neutrophil granule and cell membranes, facilitating NET release [41]. NETs then directly damage brain endothelial cells, further increasing BBB permeability. This allows more neutrophils to infiltrate the hippocampus and produce additional NETs, establishing a vicious cycle. Simultaneously, NETs induce neuronal apoptosis and activate microglia, aggravating the pathological changes associated with sepsis [147, 255]. Inhibition of PD-L1 may reduce NET release by modulating autophagy via the PI3K/AKT/mTOR pathway, offering a potential therapeutic strategy for sepsis [256] (Fig. 6).

Collectively, NETs provide a plausible bridge between systemic sepsis, BBB dysfunction, and neuroinflammation. However, because sepsis induces widespread neutrophil activation, circulating NET markers may not specifically reflect CNS-localized NET activity. Future studies should clarify whether NETs within cerebral microvessels or brain parenchyma directly contribute to cognitive dysfunction, or whether they mainly represent systemic inflammatory burden.

#### NETs in gliomas

Gliomas are primary malignant brain tumors characterized by a highly immunosuppressive and hypoxic tumor microenvironment. Tumor-infiltrating immune cells, including neutrophils, actively participate in glioma progression and therapy resistance [257]. In the glioma microenvironment, NETs formed by tumor-infiltrating neutrophils promote cell proliferation, migration, and invasion [258, 259]. Specifically, hypoxia-induced acrolein triggers the nuclear translocation of PKM2, activating HIF-1α transcription and inducing the secretion of cytokines such as IL-6 and IL-8, which, in turn, promote NET formation [259]. The binding of HMGB1, secreted by NETs, to RAGE significantly increases the phosphorylation levels of ERK1/2 and IκB, thereby enhancing IL-8 secretion [258]. IL-8, a pro-inflammatory chemokine, plays a key role in neutrophil recruitment [260]. Furthermore, IL-8 binds to CXCR2 on neutrophil surfaces, activating the PI3K/AKT pathway and stimulating the production of more ROS and NETs [258]. The newly formed NETs further alter the tumor microenvironment through these mechanisms, creating a vicious cycle that accelerates glioma development. Similarly, elevated levels of circulating NETs in the peripheral blood mediated by neutrophils are a major contributor to the poor prognosis in patients with glioblastoma [261] (Fig. 6).

In short, current evidence suggests that NETs may support tumor progression by shaping a proinflammatory and proinvasive microenvironment. However, the causal role of NETs in glioma biology remains less firmly established than in thromboinflammatory cerebrovascular disease. Future studies should determine whether NET-targeted interventions can directly alter tumor growth, immune suppression, or treatment response in CNS tumor models.

#### NETs in intracranial aneurysm

Intracranial aneurysm progression involves endothelial injury, neutrophil infiltration, and local inflammatory remodeling [262]. The occurrence and progression of intracranial aneurysms are closely associated with NETs [263]. Hemodynamic stress, tissue remodeling, and inflammation lead to endothelial damage and neutrophil activation, prompting neutrophils to catalyze H3cit through PAD4, initiating NET formation. The cytotoxic granzyme carried by NETs not only directly damages the aneurysm wall but also triggers the secretion of pro-inflammatory cytokines (e.g., ICAM-1, IL-1β, MCP-1, and TNF-α), thereby increasing the risk of aneurysm rupture [262]. Meanwhile, NET-related hub genes (e.g., TLR7, TLR2, IL1B, ENTPD4, and FPR1) are highly expressed in patients with intracranial aneurysms [264]. These genes regulate the TLR signaling pathway, promote IL-8 production, and enhance neutrophil chemotaxis, further recruiting neutrophils and inducing their transformation into NETs [264]. The risk scoring model based on these hub genes may assist in diagnosing intracranial aneurysms, and targeting NETs or related genes offers potential strategies to reduce the risk of aneurysm rupture (Fig. 6).

Overall, NET-related signatures are associated with inflammation, vascular wall injury, and rupture risk, but much of the evidence remains associative or model-based. Therefore, NETs should currently be viewed as candidate contributors to aneurysm wall instability rather than established causal determinants of rupture. Prospective studies are needed to test whether NET markers improve risk stratification beyond existing clinical and imaging parameters.

### Clearance, resolution, and physiological roles of NETs in the CNS

The role of NETs in CNS diseases depends on the balance between NET formation and NET clearance, rather than solely on whether NETs are increased [7]. In the early stage of infection, NETs capture pathogens through their DNA scaffold and concentrate antibacterial components such as histones, MPO, and NE, thereby limiting pathogen spread [3, 74]. NETs can also participate in immunothrombosis, enabling neutrophils, platelets, the complement system, and coagulation factors to form a localized defensive thrombus. However, when excessive in cerebral microvessels, this process can lead to microcirculatory obstruction and ischemic injury [265].

NET clearance typically involves nuclease-mediated cleavage of the extracellular DNA scaffold, followed by phagocytic removal [266]. DNase I and DNase1L3 can cleave the DNA backbone of NETs, causing disintegration of the reticular structure and thereby weakening the scaffolding function of NETs for thrombotic and inflammatory components [265, 266]. Nuclease-pretreated NET fragments can then be taken up and cleared by macrophages, dendritic cells, and potentially CNS-resident phagocytes, reducing the persistent exposure of the tissue microenvironment to extracellular chromatin, histones, MPO, and NE [267]. If this clearance process is impaired, residual NET components can act as a DAMP-rich scaffold that continuously activates complement, endothelial cells, inflammasomes, and thromboinflammatory responses, thereby converting a transient defense structure into a persistent source of inflammation [268].

In CNS injury, DAMPs and platelet-related signals can promote NET formation [175, 269]. Within thrombi, NETs can intertwine with fibrinogen, VWF, platelets, and red blood cells, and are associated with thrombolysis-resistant microdomains enriched in fibrinolysis inhibitors such as PAI-1 [184, 270]. This organization enhances thrombus structural stability and fibrinolytic resistance. Such stabilized thromboinflammatory scaffolds may delay thrombus resolution and reduce reperfusion efficiency. Meanwhile, persistent intravascular or thrombus-associated NETs may contribute to microvascular occlusion by enhancing fibrinolytic resistance and microcirculatory blockade [154].

Notably, NETs are not exclusively proinflammatory. Under certain highly localized inflammatory conditions, they may also participate in resolution [271]. Aggregated NETs can degrade cytokines and chemokines through NET-associated proteases, limiting further neutrophil recruitment and inflammatory spread [272]. Aggregated NETs can also bind and detoxify extracellular histones, thereby reducing histone-mediated endothelial or epithelial cytotoxicity [273]. Therefore, NET-targeted therapy should not simply aim to completely inhibit NET formation. Instead, it should seek to restore the balance of NET turnover according to disease stage, infection risk, degree of vascular inflammation, and endogenous clearance capacity.

### Central protective therapy strategies targeting NETs

Given the pathological role of NETs in various brain diseases, targeted therapies aimed at NETs have become a prominent research focus in recent years [274]. Strategies that inhibit NET formation and promote DNase I-mediated degradation, particularly through PAD4 inhibitors, have demonstrated potential for improving disease outcomes in animal models. As research progresses, several promising developments have emerged, though several critical challenges remain to be addressed (Table 1).

**Table 1:** NET-targeted therapeutic strategies

Category	Interv.	Pathways/Cytokines involved	Mechanism of action/outcomes	Ref.
Inhibition of NET formation	Cl-amidine	↑: ZO-1, VE-cadherin, Occludin, CD206, Arg-1, Bcl-2 ↓: PAD4, H3cit, MPO, Iba-1, F4/80, IL-1β, IgG, CD86, iNOs, TNF-α, p-IRE1α, p-ASK1, p-JNK, Bax, Cle-caspase-3, cGAS, STING, pIRF3, IFN-β, IL-6	M2-microglia polarization ↑; Neuron survival ↑; Anti-inflammation ↑ Neuronal death ↓; Neuroinflammation ↓; Neuronal degeneration ↓; Neuronal apoptosis ↓; Destruction of BBB ↓	[12, 203, 275]
	BB-Cl-amidine	↓: PAD4, MPO, NE, H3cit	Neurological and motor function ↑; Functional blood vessel repair ↑ Infarct volume ↓; Neuronal death ↓	[276, 277]
	GSK484	↑: p-SYK, p-AKT, CD68; Ocludin; VCL; VE-cadherin; Fermt1; MSN; TCF4 ↓: PAD4, H3cit, Cle-caspase-3, TNF-α, IL-6, IL-1β, CXCL1/2/10, CCL2/3/12	Neuron survival ↑; BBB integrity ↑; Cognitive function ↑ Neuroinflammation ↓; Neuronal apoptosis ↓	[163, 254, 278, 279]
	GW311636A	↓: PAD4, MPO, NE, H3cit	/	[276]
	Sivelestat	↑: VE-cadherin, ZO-1, Occludin, Claudin-5, IL-10, IL-4; Bcl-2 ↓: TNF-α, IL-1β, Bax, Cle-Caspase-9, Cle-caspase-3, AIM2, caspase-1, iNOS, CD86/80	Neurovascular remodeling ↑ BBB disruption ↓; Neuroinflammation ↓; Neuronal apoptosis ↓; Pro-inflammatory M1 microglia ↓	[280]
	Eda.B	↑: GSH-Px, SOD, Claudin5, Occludin ↓: MPO-DNA, H3cit, TNF-α	Neuroprotection ↑; Neurological function↑; Cerebral blood flow ↑ Infarct volume ↓; BBB permeability ↓; Neuroinflammation ↓	[281–283]
	Vitamin C	↓: ROS, MPO-DNA, H3cit	Neuroinflammation ↓; Cerebral edema ↓; Neuronal cell death ↓; Neurological dysfunction ↓	[183]
	Fucoidan	↓: P-selectin, PAC-1, αIIbβ3, MPO-DNA	Platelet activation and secretion ↓; Platelet aggregation ↓; Deep vein thrombosis ↓; Thromboinflammation ↓	[284]
	PAQ	↑: ZO-1, CD206, Arg-1, IL-10, Bcl-2, p-AMPK, Nrf2, HO-1 ↓: TUNEL, ICAM-1, MMP-2, MMP-9, F4/80, IL-1β, Iba1, CD86, TNF-α, IL-6, Bax, Caspase-3, Caspase-9	Neuroinflammation ↓; Neuronal death ↓; Cerebral edema ↓; BBB permeability ↓; Microglial inflammatory activity ↓	[94]
	HMGB1 inhibitor BoxA	↓: MPO-DNA	Microthrombosis ↓; Neurotoxicity ↓	[51]
	PD	↑: SOD, ↓: MDA, IL-6, IL-1β, TNF-α, MCP-1, MPO, NE, H3cit	Neuronal degeneration ↓; Oxidative stress response ↓; Inflammatory response ↓; Vascular permeability ↓	[285]
	BAPTA-AM	↓: PAD4, MPO, NE, H3cit	/	[276]
	Nifedipine	↓: MPO, NE, DRP1	Neutrophil ferroptosis ↓; Neuroinflammation ↓	[286]
	γ-GC	↑: ZO-1, Claudin-5, Wnt3a, β-catenin ↓: MPO, NE, H3cit	BBB integrity ↑ Neutrophil infiltration ↓; Brain infarct size ↓; Neurological deficits ↓; Cerebral edema ↓	[287]
	ASD	↑: NTSR1, cAMP ↓: PAD4	Neuroinflammation ↓	[288, 289]
	CD21	↑: p-AMPK ↓: MPO, NE, H3cit, PAD4, IL-1β, IL-17A, MMP-8, MMP-9	Neuroprotectionin ↑ Neurobehavioral deficits↓; Infarct volume ↓; Thrombosis ↓	[290]
	atRA	↑: Arg1, CD206, IL-10, VEGF, SOCS1 ↓: TUNEL, STAT1	Neutrophil N2 phenotype ↑ Neuroinflammation ↓; Infarct volumes ↓; Neurological deficits ↓	[291]
	Rovelizumab	↑: VE-cadherin, Occludin, Claudin-5 ↓: ITGB2, NOX2, IL-6, IL-1β, TNF-α, ROS, 8-OHDG, ICAM-1, Ly6G, PAD4, MMP-9, MMP-2, H3cit, MPO	Neurologic function ↑; Cerebral perfusion↑ BBB permeability ↓; Oxidative stress ↓; Inflammatory response ↓; Neuronal apoptosis ↓	[292]
	APTS	↑: CD206, IL-10, Arg-1 ↓: MPO, NE, H3cit, ROS, iNOS, H_2_O_2_, IL-6, TNF-α, cGAS, STING, NF-κB p65, caspase-1	M2-microglia polarization ↑; Neuronal survival ↑ Cerebral infarction area ↓; Neuronal apoptosis ↓; Inflammatory response ↓	[293]
	nNIP	↓: PAD4, H3cit, NE	Neurological and motor function ↑ Inflammatory response ↓	[51, 191]
Degradation of NETs	DNase I	↑: VE-cadherin, Occludin, Claudin-5, ZO-1, Bcl-2 ↓: cGAMP, cGAS, H3cit, IFN-β, IL-6, STING, pTBK1, pIRF-3, p-IRE1α, GRP78, Cleaved PARP1, Cle-caspase-3, CHOP, Bax, 8-OHDG	BBB integrity ↑; Neurological function ↑ Neurological deficits ↓; Neuronal ER stress ↓; Neutrophil infiltration ↓; Neuronal apoptosis ↓; Immunothrombosis ↓; Oxidative stress ↓	[181, 211, 294]
	rhDNase	↓: MPO, NE	Neurological function ↑; Cerebral perfusion ↑ Cerebral edema ↓	[153]
	APC	↓: Fibrinogen, VWF, PAI-1	Coagulation activity ↓; Hypercoagulability ↓	[184]
	Sivelestat	↓: Fibrinogen, VWF, PAI-1	Coagulation activity ↓; Hypercoagulability ↓	[184]

↑: upregulate; ↓: downregulate; Interv.: Intervention; Ref.: references

#### Inhibition of NET formation

Various treatment strategies that inhibit NET formation exert their effects through mechanisms such as enzyme inhibition, ROS clearance, and modulation of signaling pathways. These approaches are often associated with improvements in diseases like brain injury, inflammation, and thrombosis.

PAD4 inhibitors are important agents that target key enzyme activities, including Cl-amidine, BB-Cl-amidine, and GSK484, which demonstrate multifaceted neuroprotective potential. In murine models of TBI, Cl-amidine not only reduces the formation of NETs in the brain and peripheral blood by inhibiting PAD4 activity, but it also promotes the transition of microglia to an anti-inflammatory phenotype [203]. Mechanistically, Cl-amidine reduces the expression of IRE1α in neurons and microglia by inhibiting the STING-dependent IRE1α/ASK1/JNK signaling pathway, thereby alleviating neurodegeneration, apoptosis, and inflammatory responses [203]. Furthermore, in PAD4 gene knockout mice or following Cl-amidine administration, the levels of ZO-1, claudin-5, and occludin were upregulated, suggesting a protective effect on BBB integrity [12]. To enhance targeting and efficacy, the novel intelligent drug delivery system C-Lipo/CA enables microthrombotic binding and ROS-responsive release of Cl-amidine [275]. By inhibiting NET release, it blocks the cGAS-STING pathway, reducing levels of pIRF3, IFN-β, TNF-α, and IL-6, thus maintaining the M2 phenotype of microglia and promoting neuronal survival [275]. Similarly, BB-Cl-amidine also reduces NET formation by inhibiting PAD4 [276, 277]. Additionally, GSK484, a highly selective PAD4 inhibitor, alters the conformation of PAD4 through epigenetic regulation, leading to the loss of its protein arginine deiminase activity [163]. GSK484 not only reduces the expression of PAD4 and H3cit in the cerebral cortex and hippocampus but also significantly decreases levels of cle-caspase-3, IL-1β, IL-6, and TNF-α, reducing neuroinflammation and neuronal apoptosis [254, 278]. Notably, intraperitoneal injection of GSK484 for systemic administration may interfere with the ability of peripheral circulating neutrophils to form NETs; therefore, its impact on neutrophil capture and pathogen killing functions requires further investigation. To improve central targeting, ROS-responsive nanocarriers are more likely to penetrate the BBB and cleave upon encountering ROS, leading to the release of GSK484. This further inhibits NET formation by inhibiting PAD4, thereby exhibiting antioxidant, anti-apoptotic, and anti-inflammatory properties in TBI and middle cerebral artery occlusion injury models [279]. Interestingly, zinc supplementation has been shown to significantly reduce NET formation by lowering PAD4 expression, suggesting that maintaining a proper zinc balance is crucial for preventing excessive neutrophil activation [295]. In addition to PAD4 inhibitors, NE inhibitors also suppress NET formation by effectively blocking NE activity. For example, GW311636A can significantly reduce the extracellular DNA release of neutrophils induced by silver nanoparticles, thereby inhibiting NET generation [276]. Furthermore, a biomimetic multifunctional nanoplatform, HM@ST/TeTeLipos, has been developed, which possesses ROS-responsive and NE-inhibiting properties [280]. This platform can release the NE inhibitor sivelestat, which not only inhibits NE activity but also inactivates AIM2 inflammasomes, thereby suppressing neuroinflammation and protecting BBB integrity [280].

The generation of ROS is essential for the formation of NETs [296]. Therefore, eliminating ROS or inhibiting its production represents an important strategy for preventing NET formation. Edaravone Dexborneol (Eda. B), a potent ROS scavenger, primarily affects suicidal NETosis and is currently used in the treatment of patients with AIS [281, 282]. Treatment with Eda. B significantly reduces MPO-DNA and H3cit levels, enhances the activity of antioxidant enzymes such as glutathione peroxidase (GSH-Px) and superoxide dismutase, and increases the expression of TJs proteins, including claudin-5 and occludin, thereby preserving BBB integrity [283]. In addition to Eda. B, high-dose vitamin C administration in vivo at 500 mg/kg and in vitro at 1 μM decreases ROS generation by neutrophils and effectively inhibits NET formation in circulating blood and injured brain tissue [183]. These findings suggest that high-dose vitamin C represents a promising therapeutic strategy for modulating NET-related oxidative stress. However, the precise molecular mechanisms by which vitamin C suppresses NET formation remain to be elucidated.

The treatment that targets signaling molecules related to NETs shows comparable therapeutic efficacy. S100A8/A9, also known as calprotectin, is mainly secreted by activated myeloid cells and exerts proinflammatory and prothrombotic effects [297]. It is a structural component of NETs and functions as a signaling molecule that directly or indirectly regulates NET generation [298]. S100A8/A9 interacts with TLR4 on neutrophils. This interaction activates the ROS-NLRP3-caspase 1 pathway and subsequently induces GSDMD-dependent platelet pyroptosis [299]. The resulting platelet pyrolysis produces oxidative mtDNA, which promotes NET formation [299]. Therefore, S100A8/A9 represents a promising therapeutic target. Fucoidan is a marine polysaccharide derived from Fucus algae. Low-molecular-weight fucoidan (LMF) binds directly to S100A8/A9 and inhibits S100A8/A9-induced platelet activation, platelet recruitment, and NET formation [284]. This effect leads to measurable improvement in thromboinflammatory responses [284]. Additionally, the S100A8/A9 inhibitor Paquinimod (PAQ) blocks its interaction with TLR4 and RAGE receptors [300, 301]. It also upregulates the AMPK/Nrf2/HO-1 signaling pathway, decreases neutrophil-derived ROS, inhibits PAD4 activation, and reduces NET formation [300, 301]. Through these mechanisms, PAQ alleviates neuroinflammation and neuronal apoptosis after TBI [94]. HMGB1 is a position-dependent protein that maintains chromosomal structure and nuclear functional integrity by acting as a DNA-binding partner [302]. Once released extracellularly, it functions as a DAMP molecule and activates the NF-κB pathway through receptors including TLR4 and RAGE, thereby regulating inflammatory and immune responses [303]. Platelet-derived HMGB1 is a key promoter of NET formation [304]. It enhances NET formation through interaction with TLR2, TLR4, and RAGE [305]. However, treatment with the HMGB1 inhibitor BoxA reduces NET formation triggered by platelet-neutrophil interactions [51]. This reduction leads to a decrease in circulating MPO-DNA complexes and subsequently improves stroke outcomes [51]. S100B is highly sensitive to BBB disruption and is widely recognized as a biomarker of brain injury [306]. Polydatin (PD) ameliorates TBI-induced acute lung injury by inhibiting S100B-driven NET formation and by downregulating the expression of MPO, NE, and H3cit [285].

Various specialized treatment strategies have also been explored. Regarding the intracellular environment, the calcium chelator BAPTA-AM reduces NET formation mediated by AgNP [276]. Fluoride induces calcium imbalance in neutrophils, which leads to the opening of L-type calcium channels (LTCC). Under these conditions, extracellular free iron enters neutrophils through LTCC, triggers ferroptosis, and promotes NET release [286]. Conversely, nifedipine blocks LTCC and prevents this pathological process [286]. Regarding signaling pathway regulation, gamma-glutamylcysteine (γ-GC) increases Wnt/β-catenin signaling and subsequently upregulates the TJs-related proteins ZO-1 and claudin-5 [287]. Meanwhile, it reduces the levels of MPO, NE, and H3cit associated with NET formation, thereby helping maintain BBB integrity [287]. Akebia saponin D (ASD) is a triterpenoid saponin capable of crossing the BBB and exerting neuroprotective effects [288]. Mechanistically, ASD activates the cAMP pathway and downstream PKAc by upregulating the membrane protein neurotensin receptor 1, thereby regulating PAD4-related processes, reducing NET production after ICH, and alleviating neuroinflammation [289]. Furthermore, the phthalein derivative CD21 is a neuroprotective compound with antithrombotic activity. It may ameliorate thrombotic stroke by activating AMPK, reducing PAD4 expression, and suppressing NET formation [290]. The active metabolite of vitamin A, all-trans retinoic acid (atRA), decreases neutrophil recruitment and inhibits NET formation after stroke through its immunomodulatory effects [307]. This action is associated with increased expression of SOCS1, which suppresses activation of the JAK-STAT1 pathway [291]. However, it remains unclear whether the inhibitory effects of all-trans retinoic acid on NETs occur directly or indirectly through suppression of STAT1 signaling. Molecular blockade also provides a means of inhibiting NET formation. Rovelizumab-mediated blockade of ITGB2 reduces the expression of Ly6G, PAD4, MMP-9, MMP-2, and H3cit in brain tissue, and the content of MPO-DNA complexes also decreases [292]. It is noteworthy that nanotechnology has introduced new approaches for targeted intervention. The neutrophil hijacking nanoplatform anchors to damaged vasculature via TGase, enhancing neutrophil transport. It clears ROS and suppresses citrullinated histone pathways to redirect neutrophils from NETosis to apoptosis, thereby reducing NET formation [293].

It is worth noting that endogenous NET-inhibitory peptides are cleavage fragments of α1-antitrypsin and can specifically suppress NET formation without impairing neutrophil chemotaxis or phagocytic function [308]. The nNIF generated from the NIP sequence markedly decreases NET levels in both the circulation and the brain [191]. Through this reduction, it limits neuronal apoptosis in the affected hemisphere and prevents NET-mediated amplification of cerebral microthrombosis [51]. Additionally, it retains its inhibitory effectiveness when administered within one hour after stroke onset and provides protection against ischemic brain injury in mice of different sexes and in models with diabetes or aging [51]. Compared with PAD4 inhibitors and DNase I, nNIF shows greater therapeutic promise for ischemic stroke. Nevertheless, additional clinical studies are required to define the temporal dynamics of NET formation in stroke patients and to evaluate their efficacy in large animal models.

#### Degradation of NETs

Accelerating NET degradation offers an alternative therapeutic strategy. DNase I binds to double-stranded DNA and extracellular nuclear proteins and cleaves the DNA backbone within NET structures, thereby promoting their clearance and improving outcomes after brain injury [181, 211]. Intravenous recombinant human DNase I (rhDNase) degrades NETs in both the circulation and the CNS. This degradation reduces brain edema, improves cerebral perfusion, and enhances motor, cognitive, and psychological functions without impairing host defense against infection [153]. Further studies are needed to clarify its mechanisms in limiting edema and improving vascular function to support clinical translation. DNase I-mediated NET-targeting nanoparticles also accumulate at lesion sites through catalytic and chemotactic actions. These nanoparticles clear vascular NETs, reduce endothelial injury, and limit thrombus recruitment [294]. DNase I, APC, and sivelestat can each degrade thrombus-associated NETs, weakening their interactions with VWF and PAI-1 and reversing NET-dependent inhibition of fibrinolysis [184]. These agents also reduce platelet and endothelial activation, lower the procoagulant phenotype, and diminish deposition of VWF and PAI-1, thereby alleviating resistance to tPA [184]. Sivelestat acts mainly by inhibiting NE activity, which indirectly reduces NET formation and provides a multitarget therapeutic approach.

**Fig. 7:**
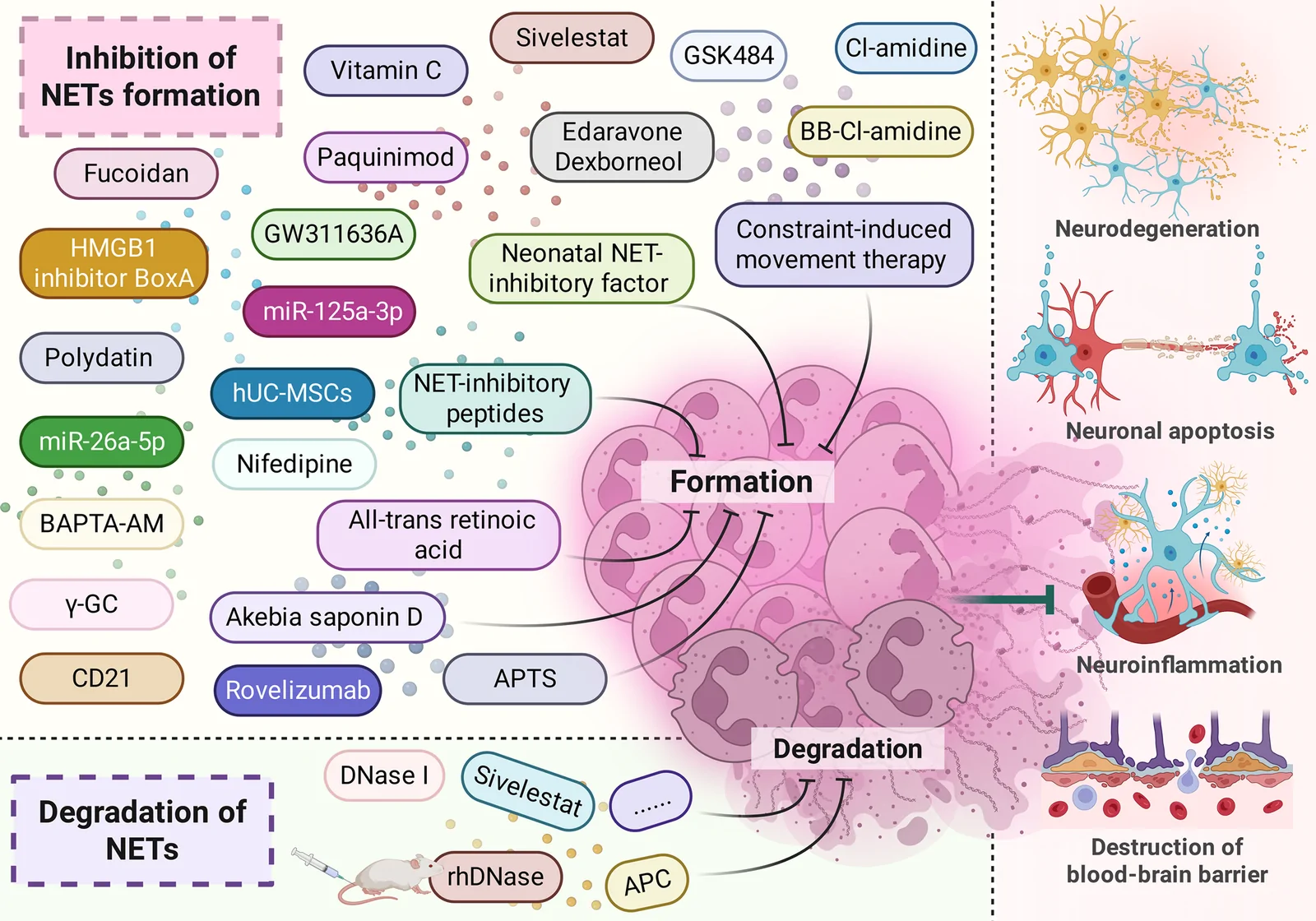
Therapeutic strategies targeting NETs. Interventions targeting NETs primarily include two approaches: inhibiting NET formation and degrading pre-formed NETs. Inhibition of NET formation involves agents such as Cl-amidine, vitamin C, GSK484, Eda.B, the HMGB1 inhibitor BoxA, and NET-inhibitory peptides. Degradation of existing NET structures relies on tools including DNase I, APC, and sivelestat. These strategies aim to mitigate NET-associated neural damage, including neurodegeneration, neuronal apoptosis, neuroinflammation, and BBB disruption

Collectively, NET-targeted therapies for CNS protection follow two main strategies: inhibition of NET formation and acceleration of NET clearance. The first strategy includes enzyme inhibition, ROS reduction, pathway regulation, and cell-based intervention. The second strategy is exemplified by DNase I and related nanotechnologies. Several of these approaches show clear neuroprotective and translational promise (Fig. 7). Future work should improve central drug targeting, ensure systemic safety, account for individual variability, and validate findings in large animal models to support the transition of NET-focused therapies from basic research to clinical application.

### Clinical translation of NET-targeted strategies in neurological disorders

Currently, no published clinical trial results have definitively demonstrated that NET‑targeted therapy can clearly improve clinical outcomes in patients with CNS disorders. However, patient sample‑based and ex vivo studies have provided a rationale for ongoing clinical trials. In AIS, neutrophils and NET components have been detected in thrombi retrieved via mechanical thrombectomy [16]. Further studies indicate that a higher NET burden within patient thrombi correlates with poorer tPA-induced thrombolysis, whereas the addition of DNase I enhances tPA-mediated thrombolysis ex vivo [164]. Furthermore, DNase I has been shown to increase the thrombolytic efficacy of tenecteplase on thrombi retrieved from AIS patients [309]. In aneurysmal SAH, elevated levels of MPO-DNA complexes in peripheral blood have been associated with delayed cerebral ischemia [310]. Additionally, various NET-related biomarkers, including H3Cit-DNA, PAD4, cfDNA, and DNase I activity, are detectable in the serum of SAH patients [311].

NET-targeted strategies currently undergoing clinical validation primarily focus on acute cerebrovascular diseases, utilizing pharmacological agents such as DNase/Dornase alfa and NE inhibitors. EXTEND-IA DNase, a phase II clinical trial, is currently evaluating intravenous Dornase alfa (recombinant human DNase 1) as an adjunctive therapy to standard reperfusion treatments in ischemic stroke with large vessel occlusion. The study aims to investigate whether this intervention improves early reperfusion prior to thrombectomy and to determine the optimal dosage for subsequent trials [312]. Similarly, NETs-target is a phase II study assessing whether the intra-procedural administration of Pulmozyme (Dornase alfa) during thrombectomy enhances arterial recanalization in patients with anterior circulation ischemic stroke who are eligible for both intravenous thrombolysis and mechanical thrombectomy; however, efficacy results have not yet been published [313]. The ReSCInD trial evaluates whether intravenous DNase I can attenuate systemic immune activation following AIS, suggesting that the clinical utility of DNase-based strategies may extend beyond thrombolysis to include the modulation of post-stroke inflammation [314]. In the context of SAH, a phase II clinical study is investigating whether daily intravenous administration of Dornase alfa for up to 14 days post-hemorrhage improves functional independence at 6 months and mitigates delayed cerebral ischemia-related injuries [315]. Beyond DNase-based therapies, sivelestat sodium is undergoing early-phase clinical investigation as an adjunctive treatment to mechanical thrombectomy for AIS with large vessel occlusion [316]. Unlike Dornase alfa, which cleaves the extracellular DNA backbone of NETs, sivelestat primarily inhibits the NET-associated effector protein NE. Consequently, it theoretically mitigates NE-mediated endothelial damage, NET formation, and thromboinflammatory responses [317, 318]. Overall, NET-targeted therapies have transitioned from mechanistic research into early-phase human trials. Nevertheless, the clinical efficacy, safety profiles, optimal therapeutic windows, and target patient populations for these strategies still require further validation through randomized, controlled, multicenter clinical trials.

### Conclusions and perspectives

In summary, NETs are context-dependent immune structures that bridge peripheral neutrophil activation with CNS inflammation, vascular injury, and thromboinflammatory damage. While excessive or uncleared NETs exacerbate neuroinflammation, BBB disruption, and microvascular thrombosis in acute neurological disorders, they act as persistent immune amplifiers in chronic conditions such as AD and MS [51, 93, 142, 167, 319, 320]. Current therapeutic strategies focus on inhibiting NET formation (e.g., via PAD4 inhibition) or promoting NET degradation (e.g., via DNase I) [184, 275, 321]. However, clinical translation faces major hurdles, including the lack of non-invasive dynamic monitoring tools, unresolved NET heterogeneity, limited safety and specificity of existing interventions, and insufficient understanding of NET interactions with other brain cell types [322–324]. Addressing these challenges will be essential for advancing precise NET modulation and developing effective neuroimmune therapies for neurological diseases. Encouragingly, several early-phase clinical trials (e.g., EXTEND-IA DNase, NETs-target, ReSCInD) are now evaluating DNase I or sivelestat in acute stroke and SAH, marking a critical step toward NET-targeted neuroprotection.

## Data Availability

No datasets were generated or analysed during the current study.

## Abbreviations

NETs: Neutrophil Extracellular Traps
CNS: central nervous system
BBB: blood-brain barrier
TBI: traumatic brain injury
SAH: subarachnoid hemorrhage
AD: Alzheimer’s disease
MS: multiple sclerosis
PMA: phorbol 12-myristate 13-acetate
PAD4: peptidylarginine deiminase 4
NE: neutrophil elastase
MPO: myeloperoxidase
TNF: tumor necrosis factor
IFN: interferon
TLRs: Toll-like receptors
PKC: protein kinase C
NOX: NADPH oxidase
ROS: reactive oxygen species
ER: endoplasmic reticulum
CDK4/6: cyclin-dependent kinase 4/6
mtDNA: mitochondrial DNA
GM-CSF: granulocyte/macrophage colony-stimulating factor
TLR4: toll-like receptor 4
SIRT1: Sirtuin 1
OPA1: optic atrophy 1
BCSFB: blood-cerebrospinal fluid barrier
BMECs: brain microvascular endothelial cells
PSGL-1: P-selectin glycoprotein ligand-1
LFA-1: leukocyte function-associated antigen-1
ICAM-1: Intercellular adhesion molecule-1
VCAM-1: vascular cell adhesion molecule-1
ChP: choroid plexus
DAMPs: damage-associated molecular patterns
PAMPs: pathogen-associated molecular patterns
CIRP: cold-induced RNA binding protein
HMGB1: high mobility group protein B1
NLRP3: NLR Family Pyrin Domain Containing 3
AIS: acute ischemic stroke
HIV-1: human immunodeficiency virus-1
PKCα: protein kinase C alpha
PKM2: pyruvate kinase M2
HIF-1α: hypoxia-inducible factor 1 alpha
METTL3: methyltransferase-like 3
VWF: von Willebrand factor
PAI-1: plasminogen activator inhibitor-1
AIM2: Absent in Melanoma 2
TREM1: triggering receptor expressed on myeloid cells 1
NINJ1: injurin1
APC: activated protein C
MS: Multiple sclerosis
ICH: intracerebral hemorrhage
MMP-9: matrix metalloproteinase 9
PAQ: Paquinimod
LTCC: L-type calcium channels
γ-GC: gamma-glutamylcysteine
ASD: Akebia saponin D
rhDNase: recombinant human DNase I
EAE: experimental autoimmune encephalomyelitis
atRA: all-trans retinoic acid
Eda. B: Edaravone Dexborneol
tPA: tissue plasminogen activator
